# Examining the Transition from Single Words to Phrase Speech in Children with ASD: A Systematic Review

**DOI:** 10.1007/s10567-024-00507-1

**Published:** 2024-11-16

**Authors:** Katherine Byrne, Kyle Sterrett, Catherine Lord

**Affiliations:** 1 Semel Institute for Neuroscience and Human Behavior, University of California, Los Angeles, USA; 2 Department of Pediatrics, University of Minnesota, Minneapolis, USA

**Keywords:** Autism, Phrase speech, Transition, Expressive language, Language development

## Abstract

“Functional speech” by 5 years of age is widely established as increasing the probability of long-term positive outcomes across a range of domains for autistic individuals. While terms such as “functional” or “useful” speech are often used, what defines these terms is not well established. Furthermore, most research focusing on language development has emphasized the transition from little or no language to use of single words, but much less is known about the transition from single words to phrase speech, which could be equally important. The verb lexicon is foundational to the development of simple, generative phrases and has been linked to prosocial behaviors and general developmental outcomes including better social communication skills, socioemotional reciprocity, and nonverbal communication in autistic children. The current systematic review synthesized information from 20 independent samples to characterize autistic children who transitioned from single words to phrase speech. On average, 48% of the pooled sample transitioned to phrase speech during the study periods. Results were highly variable across studies. Participants under the age of 5 years were more likely to transition to phrases than participants over the age of 5. Though average standard scores were above 50, children who transitioned to phrases generally demonstrated below average adaptive and cognitive skills and moderate-high ASD symptomatology. Variable measures of cognition made it difficult to ascertain patterns in cognitive skills; nonetheless, nonverbal IQ emerged as a salient predictor of the transition to phrases across studies. More research is needed to better understand who transitions beyond single words, clinical benchmarks on the way to generative phrase speech and the factors that predict this transition. Such information can be used to inform clinical decision making and develop or improve targeted interventions based on individual communication profiles. This could make the use of phrases more likely for a greater number of autistic individuals, increasing the likelihood that these individuals communicate independently and effectively with others.

**Supplementary Information:**

The online version contains supplementary material available at 10.1007/s10567-024-00507-1.

## Introduction

### Language in ASD

It is widely established that autistic individuals exhibit heterogeneous patterns of language profiles and development (Pickles et al., 2022; Tager-Flusberg, 2016; Thurm et al., 2015). Due to this heterogeneity in the language skills of autistic individuals, and to the prevalence of language delay associated with non-autistic conditions, language delay is no longer included in the most recent version of the Diagnostic and Statistical Manual of Mental Disorders (DSM-5) as a requisite for an autism spectrum disorder (ASD) diagnosis (American Psychiatric Association, 2013). In part, the decision to remove language delay from the ASD diagnostic criteria was based on the belief that the importance of language ability would more likely be recognized if language delay was noted separately from ASD diagnoses. The reality is that language delays are evident in the majority of autistic children, are often the first signs of atypical development or a plausible ASD diagnosis (Becerra-Culqui et al., 2018; Luyster et al., 2011), and may continue to affect autistic people into adulthood (Pickles et al., 2014).

Language profiles and trajectories of language development in autistic individuals vary across the lifespan to such an extent that, by school age, some individuals display advanced language skills compared to age-matched peers, while some do not develop functional expressive language, even as adults (Tager-Flusberg, 2016; Tager-Flusberg & Kasari, 2013a, 2013b). Estimates of children who remain minimally verbal (MV) after the preschool years vary based on how researchers define MV (e.g., no words vs some single words) and the age and developmental levels of the research samples involved (Koegel et al., 2020). Historically, rates of autistic minimally verbal school age children were estimated to be as high as 50% (National Research Council, 2001; Wing & Attwood, 1987). More recent studies reveal a lower, but still substantial estimate between 25 and 35%, despite efforts to increase access to intensive early interventions (Bal et al., 2020; Norrelgen et al., 2015; Pickles et al., 2014; Tager-Flusberg & Kasari, 2013a, 2013b). Some variance within the population of minimally verbal autistic individuals may be explained by co-occurring disorders of speech and language such as apraxia of speech; nonetheless, these children likely only form a small minority of nonspeaking or minimally verbal autistic children (Chenausky et al., 2020).

### Predictors of Expressive Language Development

A great deal of research has been conducted examining predictors of expressive language development. Early nonverbal skills, such as imitation, have consistently emerged as a robust predictor of later expressive language development (Anderson et al., 2007; Ellis Weismer et al., 2010; Pickett et al., 2009; Thurm et al., 2007, 2015; Wodka et al., 2013). Early gross and fine motor skills also predict the rate of language development in autistic children and early adolescents (Bal et al., 2019; Bedford et al., 2016; Iverson, 2022). Additionally, joint attention, or the ability to share attention with another person (usually around an object), has been linked to positive linguistic outcomes (Kasari et al., 2008, 2014; Mundy et al., 1990). The strength of the associations between various predictors and the development of functional language varies based on the sample being studied, such as when they were recruited, at what age the predictors were measured and at what age the outcomes were assessed. Nonetheless, they each have consistently been associated with better language outcomes.

Early language skills are another robust factor that emerge as being related to later language outcomes (Song & So, 2022). In a longitudinal cohort of over 1900 typically developing infants, language ability at age 4 predicted language ability at age 7 more accurately than a slew of individual, familial, and environmental factors, such as preterm birth, family history of language or speech delays, and maternal education (McKean et al., 2017). In studies of autistic children, having “useful” or “functional” speech by age 5 years was a prominent predictor of language in later childhood and beyond (Billstedt et al., 2007; Yoder et al., 2015). These findings further emphasize the importance of developing flexible, generative language as early as possible in development.

### “Functional” Speech and Outcomes

Children who develop “functional” speech by the age of 5 years demonstrate positive outcomes in domains other than language as well. Such long term positive outcomes include better adaptive functioning, positive well-being, higher academic achievement, vocational independence, and participation in successful social interactions (Friedman et al., 2019; Gillespie-Lynch et al., 2012; Howlin et al., 2004; Magiati et al., 2014; Mawhood et al., 2000). Though this association may be well established, the research literature in this area is ripe with amorphous terms such as “functional,” “useful,” or “communicative” speech which lack clear meaning and overlap in some ways, but not in others.

Some research studies have vaguely attempted to define these terms, such as “some speech” before 5 years, while others have attempted to define these terms more concretely ([i.e., “expressive language which is used frequently, communicatively, referentially, and in a semantically diverse manner”] Billstedt et al., 2007; Yoder et al., 2015). These imprecise and variable definitions of communicative or functional speech have existed in the autism language literature for decades and result in variable definitions of linguistic milestones across studies and results that are difficult to interpret (Koegel et al., 2020). This lack of clarity makes the study of these language transitions difficult.

### Defining “Functional” Speech

The definition that we propose to use for “functional” speech is the use of at least two-word phrases which include a noun and a verb, and which are spontaneous (i.e., not prompted), socially directed, non-rote, and used differentially across contexts (i.e., a child who says “open door” when wanting to get into a car and “open cookies” when requesting to open a bag of cookies). This definition has been used in several previous studies (Bal et al., 2019; Mouga et al., 2020; Pry et al., 2011; Thurm et al., 2015). Spontaneous, flexible phrases, particularly verb phrases, are the root of human communication. While nouns help to identify objects, people, and concepts, verbs carry the semantic meaning of a phrase and encode relationships between people and things (Hadley, 2014; Hsu et al., 2017).

Expansion of the verb lexicon is foundational to the development of simple, generative sentences (Hadley, 2006). Verbs allow individuals to talk about what has happened in the past, what is happening currently, and what will happen in the future. The use of verb phrases (such as those including “go,” “open,” or “eat”) more easily allow for individuals to communicate their needs as compared to the use of single nouns.

The onset of verb phrase use has been linked to the development of prosocial behaviors as well as general developmental outcomes, including better social communication skills, socioemotional reciprocity, and nonverbal communication in autistic children (Bal et al., 2019; Kenworthy et al., 2012; LeGrand et al., 2021). Furthermore, language milestones, such as verb phrases, have utility as indicators of prognosis because these milestones can be readily reported by parents or caregivers and screened by health professionals (Kover et al., 2016). Despite their importance, there is much that is unknown regarding why and how some children reach certain linguistic milestones, like phrase speech, and others don’t.

### Recent Efforts to Increase Language

Recent efforts have emphasized early interventions for nonspeaking children to increase their use of single words (Hampton & Kaiser, 2016; Hardan et al., 2015; Tager-Flusberg & Kasari, 2013a, 2013b). The National Institute on Deafness and Other Communication Disorders (NIDCD), a member of the U.S. National Institutes of Health (NIH), recently released a Notice of Special Interest (NOSI) encouraging researchers to submit grant proposals on minimally verbal and nonspeaking autistic individuals. While these efforts to understand more about those with very limited spoken language is important, little is known about why some autistic individuals who have some words do not develop spoken language that can be used for participation in a range of day-to-day interactions across contexts.

Thus, more information is needed about how to identify and describe the timing of when children attain phrase speech as well as the characteristics of children who make the transition to using phrases. Additionally, it will be critical to examine the predictors of this transition, and expressive language development more broadly, in order to better understand the immense individual variability of language outcomes. Such information will begin to allow us to tailor effective interventions to improve language skills and, therefore, improve outcomes across domains for autistic children (Rose et al., 2020).

### Objectives

In an effort to synthesize the current information available on children who acquire single words but do or do not yet use phrase speech, the present systematic review attempts to identify studies which included autistic participants who were either nonspeaking or were using some single words who then, at a later point, had at least some participants who were speaking in phrases. The aim of the present paper is to answer the following questions: (1) What proportion of children in the included studies developed phrase speech? (2) At what ages did those who developed phrase speech make the transition? (3) What were the cognitive, adaptive, and autism symptom profiles of the children who attained phrase speech? (4) What variables predicted the transition to phrase speech?

Based on the previous literature outlined above, we hypothesized that studies with younger participants will have greater proportions of the sample who transitioned to phrase speech as compared to studies with older participants (Pickles et al., 2014). We also hypothesized that those with greater cognitive and adaptive skills, and those with fewer or less severe autism symptom profiles would have a higher proportion of participants who transitioned to phrase speech (Ellis Weismer & Kover, 2015). Specifically, we hypothesized that nonverbal cognitive skills will likely emerge as a highly salient predictor of expressive language development and the transition to phrase speech (Thurm et al., 2007).

## Method

### Search Procedures

This systematic review was conducted in accordance with the most recent Preferred Reporting Items for Systematic Reviews and Meta Analyses (PRISMA) guidelines (Page et al., 2021) and was registered online with PROSPERO, the international prospective register of systematic reviews (registration ID: CRD42022354311). Upon consultation with Biomedical and Life Science librarians, systematic searches were conducted to identify empirical articles published between April 1966 and August 2022 that examined the transition from single words to phrase speech in autistic children. Four electronic databases were used to identify potential articles for inclusion: PubMed, ERIC, PsycINFO, and ASHAWire. Specific search terms, which can be found in Table 1, varied depending on the database used. In general, the search terms were entered in three layers. The first included terms related to autism (e.g., “ASD” and “autism”), the second included terms related to language (e.g., “language” and “communication”) and the final layer included terms related to the types of language outcomes and the longitudinal nature of the studies (e.g., “phrase speech” and “language development”). In June 2024, this search was updated to identify any additional relevant articles that have been published since the original search in August 2022.

**Table 1:** Search terms used

Database	Search terms
PubMed	(((((((((((((autism spectrum disorder[MeSH Terms]) OR (autis*[Title/Abstract])) OR (“ASD”[Title/Abstract])) OR (Asperger*[Title/Abstract])) OR (pervasive development disorder[MeSH Terms])) AND (language[MeSH Terms])) OR (communication[MeSH Terms])) OR (spoken language[Title/Abstract])) AND (language development[MeSH Terms])) OR (language growth[Title/Abstract])) OR (language acquisition[Title/Abstract])) OR (phrase speech[Title/Abstract])) OR (phrase*[Title/Abstract])) OR (single words[Title/Abstract])
ERIC	(TIAB (Autis* OR “ASD” OR Asperger* OR “Autism Spectrum Disorder” OR “Pervasive development disorders” OR “Autistic Disorder”) OR MAINSUBJECT.EXACT.EXPLODE(“Autism”) OR MAINSUBJECT.EXACT.EXPLODE(“Pervasive Developmental Disorders”)) AND (TIAB (“Spoken language” OR language OR communication OR “language development disorders” OR “language disorders”) OR MAINSUBJECT.EXACT.EXPLODE(“Language”) OR MAINSUBJECT.EXACT.EXPLODE(“Language Skills”) OR MAINSUBJECT.EXACT.EXPLODE(“Verbal Communication”) OR MAINSUBJECT.EXACT.EXPLODE(“Language Impairments”) OR MAINSUBJECT.EXACT.EXPLODE(“Communication Disorders”)) AND (TIAB (“Language growth” OR “Language acquisition” OR “Phrase speech” OR Phrase* OR “Single words” OR “language development”) OR MAINSUBJECT.EXACT.EXPLODE(“Language Acquisition”))
APAPsycINFO	(TIAB (Autis* OR “ASD” OR Asperger* OR “Autism Spectrum Disorder” OR “Pervasive development disorders” OR “Autistic Disorder”) OR MAINSUBJECT.EXACT.EXPLODE(“Autism Spectrum Disorders”)) AND (TIAB (“Spoken language” OR “language” OR “communication” OR “language development disorders” OR “language disorders”) OR MAINSUBJECT.EXACT.EXPLODE(“Language”) OR MAINSUBJECT.EXACT.EXPLODE(“Communication”) OR MAINSUBJECT.EXACT.EXPLODE(“Language Disorders”)) AND (TIAB (“Language growth” OR “Language acquisition” OR “Phrase speech” OR Phrase* OR “Single words” OR “language development”) OR MAINSUBJECT.EXACT.EXPLODE(“Language Development”))
ASHAWire	(Abstract: Autis* OR ASD OR Asperger* OR “autism spectrum disorder” OR “pervasive development disorder” OR “autistic disorder”) AND (Abstract: “spoken language” OR language OR communicat* OR “language development disorder” OR “ child language disorder”) AND (Abstract: “language growth” OR “language acquisition” OR “phrase speech” OR phrase* OR “single words” OR “language development”)

### Inclusion and Exclusion Criteria

Only articles which were peer-reviewed were included; gray literature, such as conference proceedings, dissertations, or working papers, were excluded from the initial search. Due to our specific focus on spoken language, articles which examined non-spoken communication, such as the use of augmented and alternative communication (AAC) systems (e.g., sign language) were excluded. The following predefined inclusion criteria were applied to all articles: (1) original empirical research study (e.g., no meta-analyses or review articles), (2) article published or accessible in English, (3) longitudinal design or data collected at least two times, (4) mean age of sample at first data collection timepoint less than or equal to 8 years of age, (5) sample size greater than 10, (6) participants, or a subset of participants from which data could be extracted separately, diagnosed with autism spectrum disorder (ASD; related diagnoses such as PDD-NOS or Asperger’s were included), and (7) includes a standardized measure which indicates that phrase speech was attained for the sample or a subset of the sample.

#### Assessing Phrase Speech

Initially, we searched for articles which explicitly described participants as having phrase speech or not. Surprisingly, it was rare for published articles to classify participants in this way. Instead, most articles used standardized assessments of language, such as the Preschool Language Scales (PLS; Zimmerman et al., 2011) or the Mullen Scales of Early Learning (MSEL; Mullen, 1995). As such, the authors identified scores or items on these standardized measures that indicated the child used phrase speech. On the Vineland Adaptive Behavior Scales (VABS-II and VABS-III), the selected item was: “Uses phrases with a noun and a verb” (Sparrow et al., 2005, 2016). On the PLS-3, PLS-4 and PLS-5, the selected item was: “Uses different word combinations” (Zimmerman et al., 1992, 2002, 2011). On the MSEL, the selected item was: “Uses two-word phrase” (Mullen, 1995).

After identifying specific items which indicated phrase speech attainment, we determined the lowest possible raw score that could be obtained on each measure which demonstrated proficiency on the predetermined items related to phrase speech. Next, we converted these raw scores to age equivalent scores in order to harmonize data across different measures. Thus, we yielded a minimum age equivalent for each measure that suggested phrase speech was attained. These minimum age equivalents, in addition to more general measures of phrase speech such as Autism Diagnostic Observation Schedule (ADOS) module or item-level responses on the ADOS or Autism Diagnostic Interview-Revised (ADI-R) Overall Level of Language (OLL) codes (i.e., ADOS item A1 and ADI-R item 30), were used as cutoffs to determine whether participants transitioned to phrase speech over the course of a study (Lord et al., 1994, 2000, 2012) The age equivalent cutoffs were at least 23 months on the VABS-II, 21 months on the VABS-III, 24 months on the PLS-3 and PLS-4, 25 months on the PLS-5 and 22 months on the MSEL.

We tested the validity of the determined minimum age equivalents against 15 deidentified clinical cases for whom we had item level scores. We compared ADOS OLL scores to the age equivalent score obtained on the MSEL and VABS during the same assessment period to determine whether they correspond. In other words, we tested whether those who were classified as either using phrases or not by ADOS OLL had age equivalent scores that led to the same conclusion based on the minimum age equivalent cutoffs we calculated. ADOS scores were not available for clinic cases who received the PLS. As such, we compared the PLS age equivalent scores to MSEL age equivalent scores to determine correspondence. This is reported further in the results section.

The language measures used in the included studies were those that specifically measured the use of phrase speech. Accordingly, measures which combined receptive and expressive scores, such as the Reynell Developmental Language Scales total score, or those that only measured expressive vocabulary, such as the Macarthur-Bates Communicative Development Inventory (MB-CDI), did not meet criteria for inclusion (Fenson et al., 2006; Reynell & Gruber, 1990).

### Article Review and Data Extraction

A standardized form was developed to systematically review and document whether each article in the full-text screen met specific inclusion and exclusion criteria. When an article met all inclusion criteria, the standardized form was used to extract data regarding study design (e.g., sample size, language measures used), participant characteristics (e.g., mean ages across timepoints) and study results (e.g., scores from language measures across time, portion of sample who transitioned to phrases, characteristics of participants who transitioned to phrases, etc.). Data from each of the included studies was extracted by the first and second authors independently and discrepancies were discussed and resolved together.

### Risk of *Bias*

Two risk of bias assessments were employed to document the methodological quality of the included studies. The Newcastle Ottawa Scale (NOS) was used to assess the risk of bias in nonrandomized cohort studies (*n* = 26; Lo et al., 2014). The NOS uses a star system, ranging from zero to nine stars, based on judgment among three domains: selection of the study groups, comparability of the groups, and ascertainment of the outcome of interest. Categorical ratings of studies as having a “good,” “fair,” or “poor” quality was based on total number of stars within each of the three domains named above.

The Cochrane risk-of-bias tool for randomized trials (RoB2) was used to assess the risk of bias in randomized controlled trials (*n* = 3; Sterne et al., 2019). Risk of bias was assessed in 5 domains: Randomization process, deviations from intended interventions, missing outcome data, measurement of the outcome, and selection of the reported result. Categorical ratings of overall risk of bias for each study was provided based on the ratings from each domain. Studies were rated as having a “low” or “high” risk of bias, or as having “some concerns.” The first and second authors independently assessed risk of bias in all of the included studies and discrepancies were discussed and resolved together.

## Results

### Assessing Phrase Speech

Four out of five cases on the MSEL and 4 out of 5 cases on the VABS-II were classified correctly based on ADOS OLL score. Four out of five cases on the PLS-5 were classified correctly based on MSEL age equivalent cut off.

### Study Selection

The initial search garnered 2197 articles; after removal of duplicates (*n* = 351), 1846 articles remained. All nonduplicated articles were imported into Zotero, a free and open-source citation management software. Initially, titles and abstracts were screened to exclude articles (*n* = 1585) which were clearly not relevant for the aims of this review (e.g., outcome variables not related to language, not an empirical article, study sample not diagnosed with ASD, etc.), ensuring that any potentially relevant articles were retained. After title/abstract screening, two reviewers independently completed full text scans of all remaining articles (*n* = 261). Meetings were held weekly for the reviewers to discuss inclusion/exclusion decisions for each article, reconcile discrepancies, and confirm the final selection of articles which met all inclusion criteria and which were included in the current review. Of the 261 articles which underwent full text scans, the two independent reviewers demonstrated 95% agreement on final inclusion (κ = 0.80) and 85% agreement on data extraction. Specifically, regarding discrepant data extraction between the two independent reviewers, consensus on sample characteristics (such as whether the sample had confirmed diagnoses of ASD) had to be reached for 16 articles, consensus on the language measures used had to be reached for 10 articles, consensus on the data from those who transitioned to phrases (such as age equivalents or proportions of the sample who transitioned) had to be reached for 7 articles, and consensus on whether an article was original research had to be reached for one article.

**Fig. 1:**
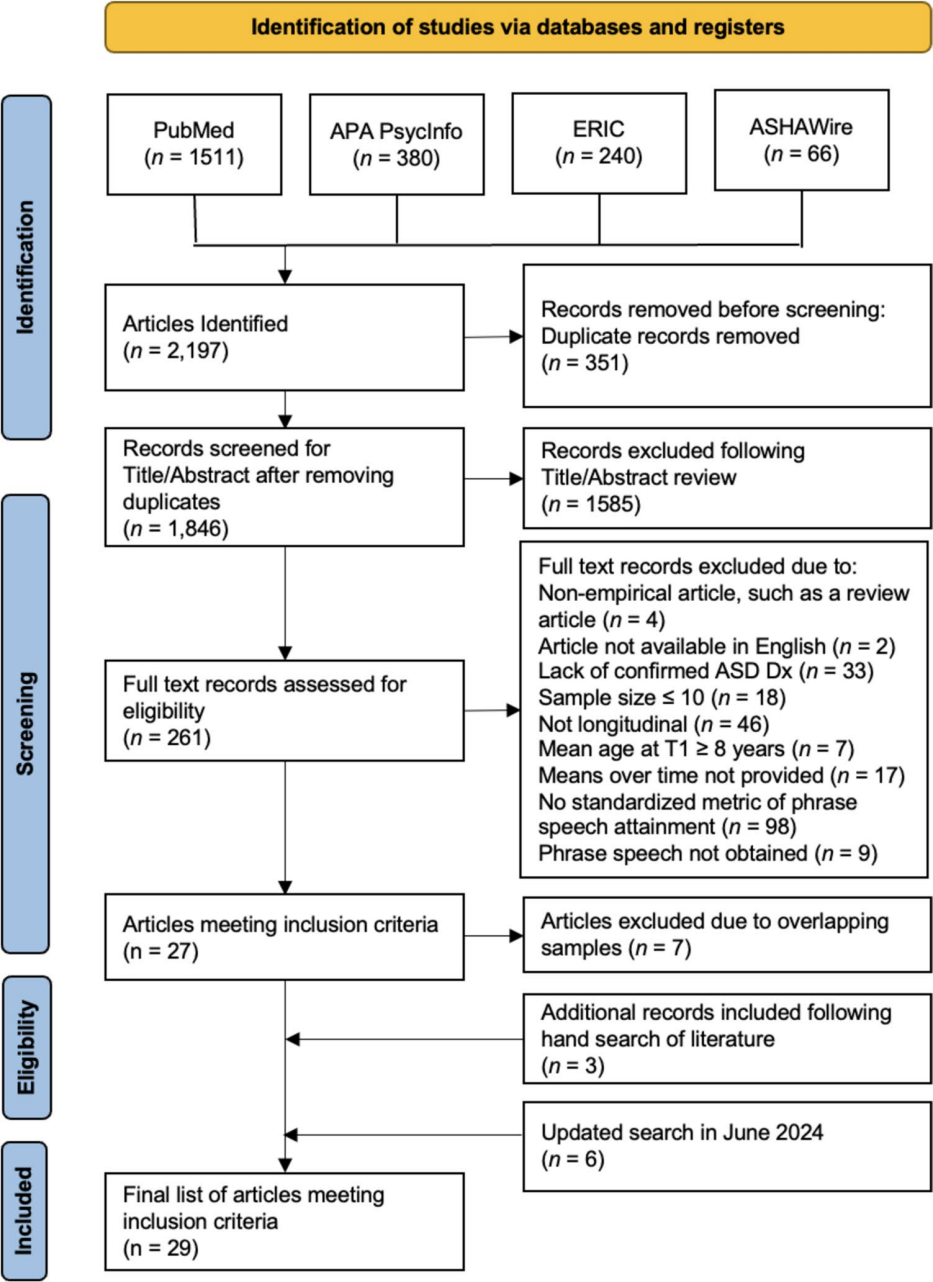
PRISMA flow diagram of study selection

Of the 261 articles remaining, 234 were excluded for the following reasons: non-empirical research (*n* = 4), article not available in English (*n* = 2), ASD diagnosis not confirmed (*n* = 33), sample size less than or equal to 10 (*n* = 18), study design not longitudinal (*n* = 46), mean age at study entry less than or equal to 8 years (*n* = 7), mean scores on standardized measures not reported in text (*n* = 17), lack of standardized metric which indicated phrase speech attainment (*n* = 98), and articles meeting all criteria above but none of the participants met the threshold of transitioning to phrase speech based on cutoffs described previously either because they began the study with too much language (*n* = 5) or never successfully transitioned from single words to phrase speech (*n* = 4). Of the 27 remaining articles which met all inclusion criteria, 14 included overlapping samples. In the case of overlapping samples, the article with the larger sample size was retained; as a result, 7 studies were excluded (Davidson & Ellis Weismer, 2017; Ellis Weismer & Kover, 2015; Haebig et al., 2013; Leonard et al., 2015; Pry et al., 2011; Siyambalapitiya et al., 2022; Zhou et al., 2019). Finally, three additional articles which met all inclusion criteria were identified by hand during literature searches and were subsequently included (Flanagan et al., 2019; Mayo et al., 2013; Paul et al., 2008). In June 2024 when the updated search for articles was completed, six additional articles were identified as meeting all inclusion criteria (Broome et al., 2023; Iao et al., 2023; Kasari et al., 2023; Kushner et al., 2023; Latrèche et al., 2024; Oosting et al., 2024). These articles were subsequently added to the final list of articles included in the current systematic review. Twenty-nine articles were included in the current systematic review. See Fig. 1 for the PRISMA flow diagram of article selection.

Additionally, a number of studies (*n* = 14) did not meet inclusion criteria but provided useful and specific information regarding the age of transition from single words to phrase speech in their sample (Goodwin et al., 2015; Grandgeorge et al., 2009; Kover et al., 2016; Lin et al., 2012; McFayden et al., 2022; Ohashi et al., 2012; Ornitz et al., 1977; Pickles et al., 2009, 2022; Pry et al., 2005; Silverman et al., 2002; Whiteley, 2004; Wickstrom et al., 2021; Xiong et al., 2024). Such studies were not included in the systematic review because of the outcome measures used (e.g., RDLS), participants included without confirmed ASD diagnosis, and ages outside of the inclusion range. Nonetheless, we decided to include a paragraph describing the ages at which these participants transitioned to phrases due to their direct connection with the aims of this research.

### Risk of *Bias*

Ratings from the NOS indicated 8 studies (28%) had “good,” 1 study (3%) had “fair,” and 17 studies (59%) had “poor” quality. Ratings from the ROB2 indicated that 2 studies assessed had “low” risk of bias and the other had “some concerns”. This information can be found in Table 2. The independent reviewers demonstrated 68% agreement on the risk of bias ratings; consensus had to be reached on six NOS ratings and one ROB2 ratings.

**Table 2:** Characteristics of included studies

Author (year)	Risk of bias rating	Sample characteristics at entry (only those diagnosed with ASD)	Age across timepoints range, if given mean (SD)	Measure used, scores across timepoints	Main findings related to phrase speech transition	Characteristics of participants who transitioned to phrases mean (SD)
Bacon et al. (2018)	Good quality	Total: *n* = 107 Early diagnosis (EDX): *n* = 69 Late diagnosis (LDX): *n* = 38 Gender, race, and maternal education not reported	EDX T1: 19.61 months (3.79) EDX T2: 35.81 months (*SD* not reported) LDX T1: 16.34 months (3.58) LDX T2: 35.04 months (*SD* not reported)	MSEL EL AE EDX T1: ~ 13 months EDX T2: ~ 26 months LDX T1: ~ 12.5 months LDX T2: ~ 28 months	On average, EDX and LDX groups transitioned to phrase speech or higher by mean age 3 years	EDX: MSEL ELC = 74.6 (9.7) VABS ABC = 83.4 (8.3) ADOS Raw Total = 19.8 (3.9) LDX: MSEL ELC = 86.8 (18.1) VABS ABC = 88.1 (12.5) ADOS Raw Total = 9.6 (5.6)
Bal et al. (2020)	Good quality	Total: *n* = 267 EDX: *n* = 86 Gender: 86% male Race: 58.1% white Maternal education: 55.3% bachelor’s degree or higher Pathways: *n* = 181 Gender: 88.4% male Race: 62.2% white Maternal education: 42.0% bachelor’s degree or higher	EDX T1: 41.56 months EDX T2: 227.88 months (12.96) Pathways T1: 37.18 months Pathways T2: 129 months (3.00)	ADOS module EDX T1: 86 participants received module 1 EDX T2: 45 participants received module 1, 41 participants moved out of module 1 Pathways T1: 181 participants received module 1 Pathways T2: 55 participants received module 1, 126 participants moved out of module 1	41 EDX participants transitioned to phrase or fluent speech by mean age 19 years; 55 Pathways participants transitioned to phrase or fluent speech by mean age 11 years	N/A—data not reported on the subset of participants who transitioned to phrase speech
Bal et al., (2019)	Good Quality	Total: *n* = 140 Verbal-Verbal (V-V): *n* = 49 Gender: 89.8% male Delayed-verbal (D-V): *n* = 46 Gender: 84.8% male Delayed-minimally verbal (D-MV): *n* = 45 Gender: 86.7% male Race and maternal education not reported	V-V T1: 3.69 years (0.07) V-V T2: 18.90 years (0.20) D-V T1: 3.41 years (0.07) D-V T2: 19.20 years (0.07) D-MV T1: 3.47 years (0.07) D-MV T2: 19.20 years (0.22)	ADI-R overall level of language (Item 30) V-V: All participants using phrases at study entry and exit D-V T1: 0% using phrases D-V T2: 100% using phrases D-MV: no participants using phrases at study entry or exit	All participants in the D-V (*n* = 46) group transitioned to phrase speech by mean age 19 years	D-V: NVIQ age 3 = 63.10 (2.19)
Barokova and Tager-Flusberg (2020a)	Poor quality	Total: *n* = 38 Gender: 81.6% male Race: 88.6% white Parent education: average 15 years of education	T1: 27.13 months (4.06) T2: 39.63 months (4.27) T3: 51.68 months (4.34)	ADOS module T1: 35 participants received module 1; 3 participants received module 2 T2: 23 participants received module 1; 15 people received module 2 T3: 12 participants received module 1; 20 participants received module 2; 6 people received module 3	Between T1 and T2, 12 participants transitioned to phrases. Between T2 and T3, 11 additional participants transitioned to phrases	N/A—data not reported on the subset of participants who transitioned to phrase speech
Bopp et al. (2009)	Poor quality	Total: *n* = 69 Gender: 84.1% male Race: 53.6% white Parent education: mothers, on average, completed some college	T1: 50 months (SD not reported) T2: 56 months (SD not reported) T3: 62 months (SD not reported) T4: 74 months (SD not reported)	PLS-3 EC AE T1: 13.96 (7.36) T2: 18.01 (8.38) T3: 21.21 (9.90) T4: 25.67 (12.19)	On average, all participants transitioned to phrase speech between T1 and T4 (mean age approximately 6 years)	CARS raw total (at study exit): 34.53 (7.83) PPVT-III (at study exit): 38.19 (29.27)
Broome et al. (2023)	Poor quality	Total: *n* = 22 Gender: 90.9% Male Race and parent education not reported	T1: 46 months (14.9) T2: 58.4 months (15.0)	PLS-4 EC AE T1: 25 months T2: 39 months	On average, all participants transitioned to phrase speech by age 5 years. Expressive language scores over time not reported across clusters	GMDS NVDQ (*n* = 13): 59.3 (23.4) Stanford-Binet NVDQ (*n* = 3): 88 (14.5) WPPSI-III NVDQ (*n* = 3): 99 (20.7) WISC-V NVDQ (*n* = 1): 86 (0.0)
Darrou et al. (2010)	Poor quality	Total: *n* = 208 Gender: 79.8% male Race and maternal education not reported	T1: 5 years (median) T2: 8 years (median)	ADI-R Overall Level of Language (Item 30; Adapted) T1: 165 participants had no speech or single words; 43 participants had at least phrases T2: 111 participants had no speech or single words; 96 participants had at least phrases	53 participants transitioned to phrases between age 5 and 8 years	N/A – data not reported on the subset of participants who transitioned to phrase speech
Flanagan et al. (2019)	Poor quality	Total: *n* = 46 Demographics not reported	T1: 56 months (3.0) T2: 66 months (4.0)	Assessment of phase of preschool language (APPL) T1: 12 participants had no speech and 6 participants had single words T2: 10 participants had no speech, 6 participants had single words, 2 participants had phrases	2 participants transitioned to phrases between T1 and T2	N/A – data not reported on the subset of participants who transitioned to phrase speech
Gengoux et al. (2019)	Low risk of bias	Total: *n* = 43 Pivotal response training-package (PRT-P): *n* = 23 Gender: 91.3% male Delayed Treatment Group (DTG): *n* = 20 Gender: 85% Male Race (of full sample): 28% white Maternal education (of full sample): 84% bachelor’s degree	PRT-P T1: 49.5 months (11.2) PRT-P T2: means not reported, approximately 55.5 months DTG T1: 47.2 months (10.0) DTG T2: means not reported, approximately 53.2 months	MSEL EL AE PRT-P T1: ~ 18 months PRT-P T2: ~ 22 months DTG T1: ~ 15 months DTG T2: ~ 17 months	On average, PRT-P group transitioned to phrase speech between T1 and T2	PRT-P: MSEL ELC: 49.9 (1.8) VABS communication SS: 63.8 (14.8) SRS-2 social communication raw: 95.8 (26.4)
Goodwin et al. (2012)	Fair quality	Total: *n* = 15* Gender: 100% male Race: 86.7% white Maternal education not reported *Only including those with ASD Dx	T1: 32.86 months (3.60) T2: 53.89 months (4.68)	MSEL EL AE T1: 20.5 months (9.69) T2: 33.0 months (19.46)	Compared ASD and TD groups. On average, ASD group transitioned to phrase speech by T2 (mean age 4.5 years)	VABS communication SS: 80.67 (18.38) VABS DLS SS: 77.07 (13.82) ADOS raw total: 13.6 (4.63)
Hellendoorn et al. (2015)	Good Quality	Total: *n* = 63* Gender: 77.8% male Race and maternal education not reported *Only including those with ASD Dx	T1: 27.10 months (8.71) T2: 45.85 months (7.16)	MSEL EL AE T1: ~ 17 months T2: ~ 42.5 months	Compared ASD and DD groups. On average, ASD group transitioned to phrase speech by T2 (mean age approximately 4 years)	MSEL Fine Motor AE: ~ 18 months MSEL Visual Reception AE: ~ 17 months
Hudry et al. (2014)	Good quality	HR-ASD: *n* = 17 Gender: 65% Male Race and maternal education not reported	T1: 7.4 months (1.2) T2: 13.7 months (1.6) T3: 23.9 months (1.2) T4: 37.7 months (3.0) Means and standard deviations provided for entire sample, doesn’t report HR-ASD separately	MSEL EL AE T1: 6.9 months (2.5) T2: 11.6 months (3.4) T3: 24.4 months (7.9) T4: 36.7 months (11.8)	Compared HR sibs with confirmed ASD dx at exit to typical, atypical but no ASD, and low risk control sibs. On average, HR-ASD sibs transitioned to phrase speech by T4 (mean age 3 years)	MSEL NV T Score: 44.0 (16.2) ADOS CSS: 6.12 (2.6)
Iao et al. (2023)	Poor quality	Total: *n* = 74 Gender: 86.5% male Race and parent education not reported	T1: 24.18 (4.44) T2: 42.74 (4.64)	MSEL EL AE T1: 10.1 months (5.95) T2: 27.3 months (11.07)	On average, all participants transitioned to phrase speech before 4 years	MSEL Overall Mental Age (at study entry): 15.20 months (4.91)
Kasari et al. (2023)	Low risk of bias	Total: *n* = 164 Discrete trial training (DTT): *n* = 82 Gender: 85% male Race: 43% white Ethnicity: 24% hispanic Maternal education: 50% bachelor’s degree or higher JASPER: *n* = 82 Gender: 82% Male Race: 39% white Ethnicity: 16% hispanic Maternal education: 41.5% bachelor’s degree or higher	DTT T1: 44.3 months (5.5) DTT T2: means not reported, approximately 49.3 months DTT T3: means not reported, approximately 56.3 months JASPER T1: 45.1 months (5.2) JASPER T2: means not reported, approximately 51.1 months JASPER T3: means not reported, approximately 57.1 months	% Using Phrases DTT T1: 0% DTT T3: 29.0% JASPER T1: 0% JASPER T3: 30.3%	By T3 (12 months after intervention began), approximately 29% of the DTT group and 30% of the JASPER group was using 3–4 word phrases	N/A—data not reported on the subset of participants who transitioned to phrase speech
Kushner et al. (2023)	Poor quality	EL-ASD Total: *n* = 12 Gender: 75% male Parent Education: parents, on average, had education beyond a High School diploma Race not reported	T1: 18 months T2: 24 months T3:36 months	MSEL EL AE T1: ~ 8 months T2: ~ 14 months T3: ~ 24 months	On average, by approximately 3 years of age, the EL-ASD group transitioned to phrase speech	MSEL receptive language T-score (at study entry): 28.4 (14.6) MSEL visual reception T-score (at study entry): 36.3 (12.7) CDI words produced: 6.7 (9.4)
Latrèche et al. (2024)	Poor quality	Total: *n* = 286 Gender: 82.5% Male Parent Education: 56.1% bachelor’s degree or higher Race or ethnicity not reported	T1: 3.6 years (0.8) T2: means not reported, approximately 4.1 years T3: means not reported, approximately 4.6 years T4: means not reported, approximately 5.1 years T5: means not reported, approximately 5.6 years	% Using Phrases Language Unimpaired (LU): By age 4.4 years, 98.4% of LU children achieved phrase speech Language Impaired (LI): By age 4.4 years, 84.6% of LI children achieved phrase speech Minimally Verbal (MV): By age 4.5 years, 17.2% of MV children achieved phrase speech	Language Unimpaired (LU): ~ 50% of LU children achieved phrase speech by age 2 years Language Impaired (LI): ~ 50% of LI children achieved phrase speech by age 3 years. Almost all reached this milestone by age 4.4 years Minimally Verbal (MV): ~ 25% of MV children achieved phrase speech by age 5 years	LU: ADOS CSS Total: 6.8 (1.7) ADOS CSS SA: 5.7 (1.8) ADOS CSS RRB: 8.5 (1.6) MSEL Composite DQ: 99.9 (11.0) MSEL Visual Reception DQ: 109.5 (16.7) MSEL Fine Motor DQ: 98.5 (16.3) LI: ADOS CSS Total: 7.2 (1.8) ADOS CSS SA: 6.2 (1.8) ADOS CSS RRB: 8.9 (1.7) MSEL Composite DQ: 67.9 (11.3) MSEL visual reception DQ: 80.6 (17.2) MSEL fine motor DQ: 73.8 (15.2) MV: ADOS CSS Total 8.6 (1.3) ADOS CSS SA: 7.6 (1.6) ADOS CSS RRB: 9.7 (0.8) MSEL Composite DQ: 33.7 (8.6) MSEL visual reception DQ: 44.7 (12.3) MSEL fine motor DQ: 46.7 (12.2)
Mayo et al. (2013)	Poor quality	Total: *n* = 119 Gender: 83.2% Male Race: 82.4% white Maternal education: No words by 18 months: *n* = 65 Gender, race and maternal education on subsample not reported	T1: 18 months T2: 52.2 months (6.1)	T1: Categorized as single words or no single words T2: MSEL EL AE T1: 65 participants with no single words @ 18 months T2: ~ 36 months	At 18 months, 65 participants were categorized as not using words. By 52 months, on average, this group had transitioned to phrase speech	No words at 18 months: MSEL Visual Reception T-score: 30.5 (14.5) MSEL fine motor T-score: 28.2 (13.3) VABS Communication SS: 67.9 (18.8) VABS DLS SS: 59.6 (10.9) ADOS CSS: 6.22 (2.42)
Mouga et al. (2020)	Poor quality	Total: *n* = 205 Became Verbal (BV): *n* = 69 Gender: 87.0% Male Always Verbal (AV): *n* = 105 Gender: 86.7% Male Never Verbal (NV): *n* = 31 Gender: 87.1% Male Race and maternal education not reported	BV T1: 42.3 months (8.3) BV T2: 88.3 months (10.3) AV T1: 54.51 months (9.05) AV T2: 91.6 months (9.04) NV T1: 49.4 months (9.3) NV T2: 86.8 months (8.3)	ADI-R Phrase Speech Item BV T1: 100 participants were classified as nonverbal at study entry BV T2: 69 participants used phrases at study exit AV: All participants using phrases at study entry NV: No participants using phrases at study entry or exit	The 69 participants in the BV group transitioned to phrases between T1 (mean age 3.5 years) and T2 (mean age approximately 7 years)	GMDS Global DQ: 70.4 (15.2) GMDS Nonverbal DQ: 85.7 (23.5) BV group transitioned to phrase speech at an average of 58.7 months (*SD* = 14.7). While the AV group reported having phrases at T1, the transition to phrases for this group occurred at an average of 36.82 months (*SD* = 9.21)
Oosting et al. (2024)	Poor quality	Total: *n* = 90 Gender: 76.7% male Race: 81% White Parent Education: 51% bachelor’s degree or higher	T1: 28 months (4) T2: 41.2 months (4) T3: 52.8 months (5)	MSEL EL AE T1: ~ 12 months T2: ~ 23 months T3: ~ 31 months	On average, all participants transitioned to phrase speech by approximately 41 months	N/A—data not reported on the subset of participants who transitioned to phrase speech
Paul et al. (2013)	Good quality	Total: *n* = 22 Milieu communication training (MCT): *n* = 12 Gender: 90.9% male Rapid motor imitation antecedent (RMIA): *n* = 10 Gender: 63.6% male Race and maternal education not reported	MCT T1: 42 months (9.6) MCT T2: means not reported, approximately 45 months MCT T3: means not reported, approximately 3–6 months following T2 RMIA T1: 51.6 months (14.4) RMIA T2: means not reported, approximately 54.6 months RMIA T3: means not reported, approximately 3–6 months following T2	VABS-II EL AE MCT T1: 9.4 months (0.6) MCT T2: 13.2 months (4.8) MCT T3: 36 months (36) RMIA T1: 10.0 months (4.8) RMIA T2: 14.4 months (7.2) RMIA T3: 19.2 months (13.2)	MCT group transitioned to phrases by approximately 4.25 years, RMIA group did not	MCT: MSEL Visual Reception AE: 22.2 (5.6) months ADOS Social Affect Raw: 14.6 (3.9)
Paul et al. (2008)	Good quality	Total: *n* = 37 “Good” language outcome: *n* = 17 “Without Good” Language outcome: *n* = 20 Gender, race and maternal education not reported	“Good” T1: 21.4 months (2.9) “Good” T2: 49.2 months (5.2) “Without Good” T1: 22.2 months (2.6) “Without Good” T2: 44.4 months (5.8)	MSEL EL AE “Good” T1: ~ 15.5 months “Good” T2: ~ 51 months “Without Good” T1: ~ 12 months “Without Good” T2: ~ 31 months	On average, all participants transitioned to phrase speech by approximately 4 years of age	“Good”: MSEL fine motor T-score: 39.1 (12.6) MSEL visual reception T-score: 44.5 (11.1) ADOS social affect raw: 10.4 (3.9) “Without Good”: MSEL fine motor T-score: 33.0 (11.4) MSEL visual reception T-score: 37.8 (11.0) ADOS social affect raw: 11.5 (2.9)
Potter et al. (2019)	Some concerns for bias	Total: *n* = 58 Sertraline: *n* = 32 Gender: 78.13% male Race: 59.38% white Maternal education: 21.88% bachelor’s degree Placebo: *n* = 26 Gender: 80.77% Male Race: 69.23% White Maternal Education: 30.77% bachelor’s degree	Sertraline T1: 51.72 months (10.8) Sertraline T2: means not reported, approximately 57.72 months Placebo T1: 44.4 months (13.2) Placebo T2: means not reported, approximately 50.4 months	MSEL EL AE Sertraline T1: ~ 18 months Sertraline T2: ~ 21 months Placebo T1: ~ 20.5 months Placebo T2: ~ 22.5 months	On average, Placebo group transitioned to phrases. Sertraline group did not	Placebo: MSEL NV DQ: 53.31 (22.25) MSEL ELC: 58.88 (14.4) VABS-II ABC SS: 68.50 (11.14) SRS-2 total raw: 101.63 (28.41)
Rose et al. (2020)	Poor quality	Total: *n* = 48 Gender: 77.1% male Race and maternal education not reported	T1: 46.88 months (*SD* not reported) T2: means not reported, approximately 58.88 months	MSEL EL AE T1: 20.76 months T2: 29.8 months	According to MSEL Item #17 (“Uses two-word phrase”), 13 participants transitioned from minimally verbal at T1 to using phrase speech at T2 (mean age approximately 5 years)	MSEL NV DQ: 50.48 (14.62) SCQ total raw: 16.67 (5.35)
Rose et al. (2016)	Poor quality	Total: *n* = 246 Gender: 82.1% male Race and maternal education not reported	T1; 44.18 months (9.44) T2: 59.29 months (7.41)	SCQ Item #1 (“Is s/he now able to talk using short phrases or sentences?”) T1: 37.5% of sample using phrases T2: 67.9% of sample using phrases	Based on the SCQ, 51 participants transitioned to phrase speech between T1 (mean age 3.7 years) and T2 (mean age 5 years)	N/A—data not reported on the subset of participants who transitioned to phrase speech
Su et al. (2021)	Poor quality	Total: *n* = 87 Gender: 75.9% male Race: 54% white Maternal education not reported	T1: 23.42 months (3.98) T2: means not reported, approximately 47.42 months	MSEL EL AE T1: 12.03 months (4.73) T2: 34.55 months (14.38)	On average, all participants transitioned to phrase speech between T1 (mean age 2 years) and T2 (mean age 4 years)	MSEL DQ: 63.73 (18.47) ADOS CSS: 8.21 (1.72)
Thurm et al. (2015)	Good quality	Total: *n* = 70 Gender: 81% male Race: 76% white Maternal education not reported MV/MV: *n* = 30 MV/PS: *n* = 17 PS/PS: *n* = 23 Gender, race and maternal education on subsamples not reported	MV/MV T1: 3.45 years (0.88) MV/MV T2: 5.44 years (0.36) MV/PS T1: 3.42 years (0.93) MV/PS T2: 5.46 years (0.39) PS/PS T1: 3.79 years (0.71) PS/PS T2: 5.46 years (0.39)	MSEL EL AE MV/MV T1: 11.73 months (4.84) MV/MV T2: 13.70 months (5.47) MV/PS T1: 15.41 months (5.33) MV/PS T2: 31.00 months (9.88) PS/PS T1: 31.36 months (6.01) PS/PS T2: 37.76 months (7.46)	Divided groups based on ADOS overall level of language (Item A1). 17 participants (i.e., MV/PS group) moved from MV to phrases by age 5.46 years	MV/PS: MSEL NV DQ: 65.17 (15.40) MSEL Verbal DQ: 40.97 (12.94) ADOS Social Affect CSS: 6.94 (1.95)
Venker et al. (2014)	Poor quality	Total: *n* = 129 Gender: 87% male Race: 86% white Maternal education: 36% had 16 + years of education	T1: 30.82 months (4.07) T2: 66.59 months (5.00)	PLS-4 EC AE T1: ~ 22 months T2: ~ 51 months	On average, all participants transitioned to phrase speech between T1 and T2. Article also reported on ADOS module transitions: 59 participants transitioned to phrase or fluent speech between T1 and T2	MSEL Nonverbal DQ: 76.39 (14.46) VABS-II DLS SS: 80.09 (9.83) ADOS Total CSS: 7.60 (1.91)
Visser et al. (2017)	Poor quality	Total: *n* = 203 Moderate-stable (M-S): *n* = 48 Gender: 83.0% male Race: 8% “nonwestern” Moderate-Improving (M-I): *n* = 10 Gender: 88.8% male Race: 10% “nonwestern” Severe-stable (S–S): *n* = 38 Gender: 73.6% male Race: 29% “nonwestern” Severe-improving (S-I): *n* = 8 Gender: 87.5% Male Race: 0% “nonwestern” Mild-stable group (*n* = 99) not reported due to not meeting criteria for ASD on the ADOS Maternal education unable to interpret	M-S T1: 31.2 months (6.0) M-S T2: 48 months (6.0) M-S T3: 66 months (9.6) M-I T1: 24 months (6.0) M-I T2: 39.6 months (4.8) M-I T3: 57.6 months (9.6) S–S T1: 30 months (6.0) S–S T2: 46.8 months (4.8) S–S T3: 62.4 months (8.4) S-I T1: 32.4 months (4.8) S-I T2: 50.4 months (4.8) S-I T3: 64.8 months (7.2)	ADOS overall level of language (Item A1; recoded*) M-S T1: 5.12 (1.3) M-S T2: 3.40 (1.4) M-S T3: 2.38 (1.1) M-I T1: 6.0 (1.2) M-I T2: 3.11 (0.8) M-I T2: 2.0 (0.0) S–S T1: 6.62 (0.8) S–S T2: 5.85 (1.2) S–S T3: 5.73 (1.6) S-I T1: 5.65 S-I T2: 3.29 S-I T3: 2.29 *Authors recoded item A1. Lower scores indicate greater expressive language skills	On average, moderate-stable, severe-improving, and moderate-improving groups achieved phrase speech by age 5.5 years. Also reported ADOS module changes across groups: in severe-stable group, 3 participants transitioned from module 1 to module 2; in severe-improving group, 6 participants transitioned from module 1 to module 2 or 3; in moderate-improving group, 8 participants transitioned from module 1 to module 2; in moderate-stable group, 29 participants moved from module 1 to module 2 or 3	M-S: ADOS-social affect total raw: 12.21 (2.5) NVIQ: 71.6 (18.2) M-I: ADOS-social affect total raw: 13.00 (3.3) NVIQ: 83.0 (15.2) S–S: ADOS-social affect total raw: 18.54 (1.4) NVIQ: 45.3 (15.9) S-I: ADOS-social affect total raw: 17.38 (1.7) NVIQ: 60.7 (13.1)
Zwaigenbaum et al. (2016)	Poor quality	Total: *n* = 103 Gender: 68.9% Male Diagnosed at 18 months: *n* = 19 Diagnosed at 24 months: *n* = 27 Diagnosed at 36 months: *n* = 47 Race and parental education not reported	T1: 2 years T2: 3 years	MSEL EL AE Dx @ 18 months T1: ~ 16 months Dx @ 18 months T2: ~ 27 months Dx @ 24 months T1: ~ 20 months Dx @ 24 months T2: ~ 29 months Dx @ 36 months T1: ~ 23 months Dx @ 36 months T2: ~ 33 months	On average, participants in the Dx at 18 months and Dx at 24 months groups transitioned to phrases between two and three years of age. Participants who were diagnosed at 36 months already had phrase speech by two years of age	Dx @ 18 months (scores obtained from 2-year evaluation): MSEL Visual Reception T-score: 42.9 (15.1) MSEL fine motor T-score: 39.9 (14.6) ADOS Total CSS: 6.6 (2.9) Dx @ 24 months: MSEL visual reception T-score: 44.5 (19.2) MSEL fine motor T-score: 38.2 (15.6) ADOS total CSS: 7.0 (2.1)

*AE* age equivalent, *EC* expressive communication, *EL* expressive language, *MSEL* mullen scales of early learning, *ELC* early learning composite, *DQ* developmental quotient, *PLS-3* preschool language scales, third edition, *PLS-4* preschool language scales, fourth edition, *PLS-5* preschool language scales, fifth edition, *VABS-II* vineland adaptive behavior scales, second edition, *VABS-III* vineland adaptive behavior scales, third edition, *ABC* adaptive behavior composite; *DLS* daily living skills, *NVIQ* nonverbal intelligence quotient (scaled score with mean of 100 and standard deviation of 15), *SS* standard score (scaled score with mean of 100 and standard deviation of 15), *ADOS* = autism diagnostic observation schedule, *CSS* calibrated severity score, *GMDS* Griffiths mental development scales, *SCQ* social communication questionnaire, *WPPSI-III* Wechsler preschool and primary scale of intelligence, third edition, *WISC-V* Wechsler intelligence scale for children, fifth edition, *CDI* Macarthur–Bates communicative developmental inventory

For consistency, reported scores on standardized instruments were converted to age equivalents, whenever possible. Missing standard deviations indicate that it was not provided in the corresponding manuscript

### Characteristics of Included Studies

Table 2 describes the characteristics of all studies included in the current systematic review, including the sample characteristics (e.g., sample size, age), language measures used, main findings in relation to phrase speech transition, and characteristics of the participants who transitioned to phrases.

#### Measures

Fifteen (52%) studies used the MSEL expressive language subdomain, 5 (17%) used item-level responses from the ADI-R, ADOS, or Social Communication Questionnaire (SCQ; Rutter et al., 2003), 2 (7%) used ADOS module, 3 (10%) used the PLS expressive communication subdomain, 1 used binary categorizations of phrase speech based on observation (3%), 1 used categorizations set forth in Tager-Flusberg et al., (2009 [3%]), 1 (3%) used VABS expressive language subdomain, and 1 (5%) used the Assessment of Phase of Preschool Language (APPL) to characterize participants’ language.

#### Characteristics of Sample who Transitioned to Phrases

Of the 29 included studies, six (21%) did not provide any descriptive information regarding the subset of participants who transitioned from single words to phrase speech throughout the course of the study. Of the 23 studies which did, demographic information on gender, race, and/or caregiver education can be found in Table 1.

##### Age of Transition

Across all included studies, 1389 participants (46.5%) transitioned from single words to phrase speech. Only one study reported the age at which phrases emerged for their sample; the mean age was 58.7 months (*SD* = 14.7; Mouga et al., 2020).

Next, we looked specifically at studies which reported on language data for children more than 5 years of age. On average, samples with a mean age greater than 5 years had lower proportions of participants who transitioned to phrase speech, as compared to samples at or below 5 years. After 5 years of age, all studies reported less than 50% of the sample transitioning to phrase speech [with the exception of one subgroup of participants in Visser et al., (2017)]. More specifically, the mean proportion of samples who transitioned to phrase speech after 5 years of age was approximately 30% across studies compared to more than 50% before 5 years of age.

##### Cognitive Scores

Twenty-one articles (72%) provided cognitive information using a variety of measures for the subset of participants who transitioned to phrase speech. Seventeen studies used the MSEL, three studies reported nonverbal IQ (NVIQ) using a variety of tests based on the age of the participants, and one study used the Griffiths Mental Development Scales (GMDS; (Huntley, 1996). Across all measures, average NVIQs within the samples ranged from 59 to 88.0 (mean = 73.3, *SD* = 12.0). MSEL Early Learning Composite scores, a global score of cognition including verbal and nonverbal skills, ranged from 49.9 to 86.8 (mean = 67.5, *SD* = 16.4), and MSEL nonverbal developmental quotients (DQ; a standard score which is calculated by dividing the child’s developmental age by chronological age and multiplying by 100) ranged from 50.5 to 76.3 (mean = 61.3, *SD* = 9.4).

Next, we examined trends in cognitive scores for those who transitioned to phrase speech. Surprisingly, we were unable to ascertain consistent patterns of cognitive skills based on the age at which samples transitioned to phrase speech. There was considerable variability in the cognitive profiles of the participants, such that some participants who transitioned to phrase speech before the age of 5 had lower cognitive scores than some participants who transitioned to phrase speech after the age of 5. This is discussed in more detail in the discussion.

##### Adaptive Skills

Six articles (21%), all which used the VABS, provided adaptive skill information across a range of domains for the subset of participants who transitioned to phrase speech. Two studies provided global scores of adaptive skills (i.e., the Adaptive Behavior Composite [ABC]), which describes adaptive behaviors across domains of communication, daily living skills, and socialization. Average scores ranged from 68.5 to 88.1 (mean = 80.0, *SD* = 10.2). Three studies provided information on the communication domain; scores ranged from 63.8 to 80.7 (mean = 70.8, *SD* = 8.8). Two studies provided information on the daily living skills domain; scores ranged from 59.6 to 80.1 (mean = 72.3, *SD* = 11.1). The distribution of scores for adaptive behaviors was too variable to identify any consistent patterns. Generally, however, adaptive scores related to the cognitive scores of the participants (i.e., participants who had lower cognitive scores also tended to have lower adaptive scores).

##### ASD Symptoms

Fourteen articles (48%) provided information on ASD symptoms for the subset of participants who transitioned to phrase speech. Eleven articles used the ADOS; 4 used the ADOS total Calibrated Severity Score (CSS), 3 used the social-affect raw score, 2 used the total raw score, and 1 used the social affect CSS. ADOS total CSS ranged from 6.1 to 8.6 (mean = 7.0, *SD* = 1.0). ADOS total raw scores ranged from 9.6 to 19.8 (mean = 14.3, *SD* = 5.14). ADOS social-affect raw scores ranged from 10.4 to 14.6 (mean = 12.3, *SD* = 1.6). The only reported average social-affect CSS score was 6.94. Two articles provided information on ASD symptoms using the SRS; one provided the total raw score (mean = 101.63, *SD* = 28.4), and one provided the social communication raw score (mean = 95.8, *SD* = 26.4). One article provided information on ASD symptoms using the SCQ total raw score (mean = 16.67, *SD* = 5.4). With the exception of some outliers, such as those in Bacon et al. (2018) who transitioned to phrases by 3 years but were identified as receiving an early ASD diagnosis at approximately 19 months, a general trend was identified such that those who transitioned to phrase speech at earlier ages were more likely to have lower ASD symptoms.

##### Predictors of Transition

Six articles (30%) provided information on predictors of functional language, expressive language growth, or transitions to phrase speech.

*General Verbal and Nonverbal Cognition.* Mouga et al. (2020) found that the probability of belonging to the “became verbal” subgroup (defined as having phrase speech), as compared to the “never verbal” subgroup, increased as global DQ and nonverbal DQ increased. Specifically, the children with the greatest likelihood of developing phrase speech were those that had a global DQ greater than 62.5 and a nonverbal DQ greater than 73.5. Similarly, Thurm et al. (2015) found that both verbal and nonverbal DQ at age 3 years predicted expressive language skills at 5 years. Moreover, each percentage increase in verbal DQ precipitated a 3% increase in the likelihood of developing phrase speech by study exit.

A study conducted by Paul et al. (2008) found that those with “good” language outcomes (characterized by a VABS expressive language age equivalent of 30 months) were more likely to have stronger nonverbal cognition as measured by the MSEL. After controlling for nonverbal cognitive scores, those with “good” language outcomes also had higher receptive language skills, were more likely to respond to joint attention on the ADOS, engage in symbolic play, produce more words and sounds on the Communication and Symbolic Behavior Scales—Developmental Profile (CSBS-DP), and demonstrate fewer stereotyped or repetitive interests and behaviors on the ADOS, as compared to the group of children who did not have “good” language outcome.

*Other Predictors.* Su et al. (2021) found that early social motivation was predictive of later functional language use. Hellendoorn et al. (2015) revealed how early fine motor functioning, mediated by object exploration and visuospatial cognition, was predictive of later expressive language skills in autistic children. Rose et al. (2020) examined how object play, visual attention, and symbolic word learning influenced language growth. Results demonstrated that functional use of objects in play at approximately 4 years of age was the only variable predictive of expressive language growth. Furthermore, participants who developed phrase speech by study exit had higher nonverbal cognition and lower ASD symptomatology at study entry compared to those who remained minimally verbal (defined here as using up to 8 single words but not yet using two-word phrases).

*Who Didn’t Transition?* In order to ease comparison, we are including summary data from four studies which included participants who did not have phrase speech at study entry and, by study exit, never successfully transitioned to using phrases (Manwaring et al., 2019; McFayden et al., 2024; Swanson et al., 2017; Walton & Ingersoll, 2016). The ages of the final visit in each study in which phrase speech was not obtained ranged from 24 to 42 months. Manwaring et al. (2019) and Swanson et al. (2017) reported mean MSEL Nonverbal Developmental Quotients (NVDQ) of 60.3 and 87.8, respectively. Walton and Ingersoll (2016) reported an average NVDQ of 52.2 from the Bayley Scales of Infant Development (BSID-III; (Brady, 2006). Finally, McFayden et al. (2024) reported a 24 month receptive language age equivalent of approximately 19 months. None of the studies reported on adaptive skills. Three of the studies reported autism symptom severity scores. Swanson et al. (2017) reported an average ADOS CSS of 5.85, Walton and Ingersoll (2016) reported an average ADOS Raw Total Score of 15.32, and McFayden et al. (2024) reported average ADOS CSS scores of 6 in the Social Affect domain and 6.46 in the Restricted and Repetitive Behaviors domain.

##### Additional Data

Fourteen studies not included in the current review provided data on the age at which participants in their sample transitioned to phrases (Goodwin et al., 2015; Grandgeorge et al., 2009; Kover et al., 2016; Lin et al., 2012; McFayden et al., 2022; Ohashi et al., 2012; Ornitz et al., 1977; Pickles et al., 2009, 2022; Pry et al., 2005; Silverman et al., 2002; Whiteley, 2004; Wickstrom et al., 2021; Xiong et al., 2024). The ages ranged from 21.3 months to 56.5 months (mean = 36.8 months, *SD* = 9.7). A study conducted by Wickstrom et al. (2021) retrospectively asked 479 parents of autistic children or children with genetic abnormalities associated with autism when their children had acquired phrase speech. The majority (84%) of the sample acquired phrases before age 7, 1% of the sample acquired phrases between 7 and 9.5 years of age, and 10% of the sample had not yet acquired phrases by the age of 9.5 years (Wickstrom et al., 2021). Xiong and colleagues (2024) collected data on linguistic milestones from 610 autistic children. Results demonstrated that 34.5% of children used two- or three-word phrases by 2 years, 55.4% by 3 years, 72.0% by 4 years, and 77.7% by 5 years. Another less recent study determined that, from a sample of 222 autistic children, only 20% were combining words into three word phrases by 5 years (Pry et al., 2005).

## Discussion

### Age at Transition to Phrases

Of the studies reviewed, the average age of phrase speech development occurred at 36 months. Yet, it is important to note that there was considerable individual variability in the age at which participants transitioned to phrase speech. One study of 228 autistic children, the majority of whom had a developmental quotient less than 50, found that between the ages of 5 to 8 years, 25% (*n* = 53) of the sample transitioned from either using no words or some single words to phrase speech (Darrou et al., 2010). Other research conducted with children with ASD and serious language delays showed that phrase speech can develop after the age of 5 years (Wodka et al., 2013). However, a systematic review on speech acquisition in older autistic children over the age of 8 did not find evidence of developing phrase speech after the age of 13 years (Pickett et al., 2009). While the possibility of developing phrases in later childhood is promising, it is important to remember that earlier age of speech acquisition is predictive of more positive prognosis across domains (Mayo et al., 2013). The younger children are when they begin early intervention, the better their outcomes tend to be, though more information on intervention intensity is needed (Guthrie et al., 2023). This highlights the need for ongoing intensive interventions tailored to the individual’s linguistic profile and aimed at speech development before, but also continuing after the age of 5 years.

Of the small number of studies (*n* = 4) in which participants did not transition to phrases, studies concluded when participants were between the mean ages of 2 to approximately 3.5 years. Thus, it is difficult to know whether these participants would have transitioned to phrases had data collection continued. More longitudinal research that continues into early childhood is necessary to better understand who does and does not transition to phrase speech past the age of 5.

### Cognitive and Adaptive Skills in Transition to Phrases

While the profiles of cognitive and adaptive skills were quite variable among the group of participants who transitioned to phrase speech, some key takeaways can be drawn, nonetheless. Full scale cognitive scores ranged from 50 to 87 and nonverbal cognitive scores ranged 50 to 88. Adaptive skills standard scores in the areas of communication, daily living, and socialization ranged from 68 to 88. It is clear that the overwhelming majority of children who transitioned to phrases were delayed in both cognitive and adaptive skills regardless of the age at transition. Even those who scored highest on assessments of cognitive and adaptive skills received scores, on average, in the low-average range. It is important to acknowledge that adaptive skills, IQ and language are often highly correlated early in development; nonetheless, delayed cognitive and adaptive skills are unsurprising, given the extensive literature documenting these delays in samples of autistic individuals (Paul et al., 2004; Pugliese et al., 2016).

However, while the participants who transitioned to phrases were delayed to some degree, it is noteworthy that they consistently demonstrated verbal and nonverbal cognitive scores greater than 50. This finding has been replicated elsewhere—a review examining speech acquisition in older, nonverbal individuals with ASD found that speech acquisition after the age of 5 was more likely to occur in individuals with IQs greater than 55 (Pickett et al., 2009).

It was surprising to see that those who transitioned to phrase speech at earlier ages did not consistently show higher cognitive scores as compared to those who transitioned to phrase speech later. For example, in Bacon et al. (2018), participants in the early diagnosis group who transitioned to phrases before 3 years had a full scale IQ of approximately 75. Participants in Mouga et al. (2020) transitioned to phrase speech by 7.5 years and had global developmental quotients of 70. It is possible that this can be attributed, at least in part, to differences in measurement (i.e., use of the MSEL versus the GMDS or reporting different aspects of cognition, such as verbal versus nonverbal scores). Yet, as noted in Latréche et al. (2024), the language unimpaired group who transitioned to phrases the earliest did demonstrate greater nonverbal cognitive skills as compared to the language impaired and minimally verbal group, with the minimally verbal group having the lowest nonverbal cognitive scores. Nonetheless, the lack of consistent pattern from some studies in the current review is noteworthy, considering the expectation that lower age at phrase speech attainment would be related to higher cognitive scores.

While it is difficult to make comparisons due to the small number of studies which reported that their sample did not transition to phrase speech (*n* = 4), nonverbal cognitive scores were generally lower compared to those who did transition to phrases. For example, Manwaring and colleagues (2019) reported MSEL NVDQs of approximately 60. It is important to note that the study conducted by Swanson and colleagues (2017) stopped data collection at 24 months and, had data collection continued, it is reasonable to assume that some of their sample may would have transitioned to phrases given the relatively higher nonverbal cognitive scores.

Among those who transitioned to phrase speech, average scores of ASD symptoms were high. The average ADOS total raw score was 14 and CSS score was 7, which corresponds to symptom severity in the moderate-high range. Findings regarding the role of ASD symptoms in the transition to phrase speech are mixed. One study found that CSS scores were not predictive of expressive language growth (Thurm et al., 2015), while another found that those who transitioned to phrase speech had lower ASD symptoms compared to those who did not transition to phrase speech by study exit (Rose et al., 2020). More prospective research in this area is warranted to discern how whether autism symptoms specifically, in addition to global measures of cognition, are related to trajectories of language development and linguistic outcomes.

### Predictors of Transition to Phrase Speech

The relationship between nonverbal cognition and expressive language is complex, given that some children display greater nonverbal abilities than verbal (Pecukonis et al., 2019). Nonetheless, consistent with our hypotheses, greater nonverbal cognition emerged as the most salient predictor of the transition to phrase speech. All articles included in this review which measured nonverbal skills came to similar conclusions regarding the positive predictive power of nonverbal cognition in relation to expressive language. While a small number of articles (not meeting criteria for the current review) have not found this relationship to hold (Chenausky et al., 2018; Girolamo & Rice, 2022), it has been widely established in other independent samples of individuals with ASD (Anderson et al., 2007; Luyster et al., 2008; Pecukonis et al., 2019), including children with stronger verbal abilities (Brignell et al., 2018) and those with severe language delay (Wodka et al., 2013).

One study found that the influence of nonverbal cognition on expressive language skills was mediated by object exploration and visuospatial cognition (Hellendoorn et al., 2015). Iao et al. (2023) found that motor imitation was a strong predictor of concurrent and longitudinal measures of expressive language. Other studies have linked expressive language to fine motor skills (Butler & Tager-Flusberg, 2023; LeBarton & Iverson, 2013). It is possible that those with higher nonverbal skills are more likely to explore their surrounding environment or engage with objects in ways that beget increased opportunities for language use. However, it is unclear whether the predictive value of nonverbal cognition in this study can be accounted for by earlier verbal scores. Considering that only one article in the current review examined object exploration, and most of the articles which measured nonverbal cognition did not look at fine motor and visual spatial skills separately, more detailed explorations of the individual components of nonverbal cognition in relation to expressive language development are warranted. It will be necessary for researchers to systematically examine these predictors with greater precision to better understand the nature of the relationship.

Lastly, the relatively small number of studies which met inclusion criteria for this review should be noted. Of the almost 300 studies that received full-text screens, only 29 articles were identified with longitudinal data on independent samples who transitioned from single words to phrase speech. While the transition to phrase speech seems like an important linguistic milestone in working towards “functional” language across contexts, only eleven studies explicitly reported the degree to which the transition to phrase speech occurred throughout the course of the study (though it is important to note that there were diverse research aims and outcome variables across studies). Practical linguistic milestones in research are rarely reported and, thus, meaningful transitions between milestones may go unnoticed (Tager-Flusberg et al., 2009). It is important for future research to consider the practical implications of reporting linguistic milestones, as milestone attainment across individuals may provide clinically useful information that will further our understanding of language development in an incredibly heterogeneous group of individuals.

### Limitations

A number of methodological limitations should be mentioned. While it was necessary to use a systematic method in determining whether phrase speech was reached, the method of determining cut-off age equivalents across the various language measures and the use of group means may have led to errors. It is likely that there were participants who transitioned to phrases who were missed, or participants who did not transition to phrases who were mischaracterized because we were looking at average age equivalents across samples. Furthermore, the MSEL and PLS items pertaining to use of phrases does not require that a verb be included. While we tried to address this imperfect method of measuring phrase speech using standardized assessment scores by testing the validity of the age equivalent cut off scores against the ADOS OLL code and other measures, language status and milestone attainment may not correspond entirely to scores on standardized assessments (Thurm et al., 2015). Because the use of verbs is an important linguistic milestone related to expressive language development, standardized measures alone may not be sufficient in characterizing phrase speech and would require the use of natural language samples.

In some cases, multiple standardized language measures within the same article characterized participants in different ways. For example, in Hardan et al. (2015), all participants scored above the cut-off of phrase speech at study entry according to the PLS-4 (indicating that they had phrases at study entry), while the same participants were considered to have transitioned to phrases only when using the VABS-II. While we did not include studies in which different measures yielded discrepant conclusions about group status, these contrasting scores highlight the complicated nature of measuring expressive language. While there are proposed “best practices” of measuring language skills, such as the use of natural language samples (Barokova & Tager-Flusberg, 2020b), there currently exists no agreed upon battery or “gold standard” of measuring expressive language skills or even agreement in terminology. Different measures result in different scores and, therefore, different profiles of language skills. It is imperative to consider the types of measures used (e.g., parent report questionnaire, observational assessment conducted by trained assessor, etc.) when interpreting scores or when comparing results across studies, even when using standardized metrics such as standard scores or age equivalents.

Next, independent ratings of risk of bias had low percent agreement. However, authors came together to consensus these ratings to ensure they were appropriate.

Lastly, we recognize that focusing solely on spoken expressive language does not provide a comprehensive representation of overall communication skills, including the use of AAC or signs for nonverbal individuals. Nonetheless, the aims of the current review were to better understand the transition from spoken single words to phrase speech and, as such, the decision was made to focus on spoken expressive language.

### Next Steps

More research in this area is needed to better understand who does and who does not move beyond the use of single words and into flexible, generative phrase speech. Much of the previous research has examined varying types of language outcomes (e.g., expressive vocabulary, composite expressive and receptive language, etc.) and used a variety of data collection methods (e.g., natural language sampling, parent report, clinician administered assessment). The use of varying outcome measures and, oftentimes, the complete absence of mean scores reported in articles, prevents comparisons across samples and limits clinical interpretability.

It has been recommended that published articles include “fine-grained” data, such as item-level analyses indicating language milestone attainment, to provide detailed and easily interpretable information regarding specific language level and use (Rose et al., 2016). The use of empirically driven agreed-upon standards for benchmarks of “functional” language, such as those put forth by Tager-Flusberg et al. (2009) would allow for easily interpretable results which are comparable across samples, and which better characterize individual variability in spoken language compared to the use of global scores derived from standardized assessments.

## Conclusion

It has been widely documented that “functional” use of language by the age of 5 is related to more positive outcomes (Billstedt et al., 2007; Howlin et al., 2004). However, researchers have historically struggled to develop consistent and clear definitions of desired linguistic levels, making functional speech difficult to measure. The flexible use of phrases across contexts, particularly verb phrases, is foundational to language development and true reciprocal communication (Hsu et al., 2017). Yet, close examination of the transition from single words to phrase speech for children with ASD has largely gone unexamined.

The ability to generate flexible phrases or sentences has tremendous implications for social communication, creation and maintenance of social relationships and for independence. If researchers and clinicians are better able to understand what predicts the transition from single words to phrase speech and, eventually, to fluent speech, informed clinical decision making and targeted interventions can be improved based on individual communication profiles. As such, the transition to communicative, generative speech may become possible for a greater number of children with ASD and, therefore, improve outcomes across a range of related domains.

## Supplementary Information

Below is the link to the electronic supplementary material.

Supplementary file1 (PDF 137 KB)

Supplementary file2 (DOCX 58 KB)
