# Supplementary material for: Examining the Transition from Single Words to Phrase Speech in Children with ASD: A Systematic Review
Source: Clin Child Fam Psychol Rev. 2024 Nov 16;27(4):1031–53. doi: 10.1007/s10567-024-00507-1 (PMC11609125; doi:10.1007/s10567-024-00507-1)
Supplement: Supplementary file 1 — Supplementary file1 (PDF 137 KB) [file 10567_2024_507_MOESM1_ESM.pdf]

Phrase Speech Systematic Review

1. Who is filling out this form?

____

2. Citation?

____

3. Year published?

____

4. Is this study original research?

Mark only one oval.

- ( ) Yes
- ( ) No

5. Is this study published in English?

Mark only one oval.

- ( ) Yes
- ( ) No

6. Do participants enrolled in the study have a primary diagnosis of ASD? (NOTE: If not all participants have ASD, must at least be able to extract the data from the ASD sample)

   Mark only one oval.

   - Yes
   - No

7. Is the sample size of autistic individuals with complete language data greater than 10?

   Mark only one oval.

   - Yes
   - No

8. If yes, what is the n?

   ____

9. Does the study collect at least one measure of language over time (i.e., at least two timepoints)?

   Mark only one oval.

   - Yes
   - No

10. Is the mean age at the first timepoint equal to or less than 8 years of age?

    Mark only one oval.

    - Yes
    - No

11. What kind of study is this?

Mark only one oval.

- ( ) Longitudinal Cohort
- ( ) Pre-post intervention
- ( ) Infant Sib
- ( ) Other: ____________________

12. Is there a standardized measure that indicates attainment of phrase speech which is collected at least two times?

Mark only one oval.

- ( ) Yes
- ( ) No

13. What measure(s) was used?

____

14. What score was collected (T, AE, Raw, SS)

Mark only one oval.

- ( ) T
- ( ) AE
- ( ) Raw
- ( ) SS
- ( ) Other: ____________________

15. What timepoints/ages was the measure(s) collected at?

16. G1 - Age at T1

17. G1 - Age at T2

18. G1 - Age at T3

19. G1 - Score at T1

20. G1 - Score at T2

21. G1 - Score at T3

22. G1 - AE at T1

23. G1 - AE at T2

24. G1 - AE at T3

25. G2 - Age at T1

26. G2 - Age at T2

27. G2 - Age at T3

28. G2 - Score at T1

29. G2 - Score at T2

30. G2 - Score at T3

31. G2 - AE at T1

32. G2 - AE at T2

33. G2 - AE at T3

34. G3 - Age at T1

35. G3 - Age at T2

36. G3 - Age at T3

37. G3 - Score at T1

38. G3 - Score at T2

39. G3 - Score at T3

40. G3 - AE at T1

41. G3 - AE at T2

42. G3 - AE at T3

43. G4 - Age at T1

44. G4 - Age at T2

45. G4 - Age at T3

46. G4 - Score at T1

47. G4 - Score at T2

48. G4 - Score at T3

49. G4 - AE at T1

50. G4 - AE at T2

51. G4 - AE at T3

52. Was phrase speech attained according to the standardized measure?

Mark only one oval.

- Yes
- No
- Other:

53. Additional notes from this citation?

This content is neither created nor endorsed by Google.

Google Forms
