# Supplementary material for: Examining the Transition from Single Words to Phrase Speech in Children with ASD: A Systematic Review
Source: Clin Child Fam Psychol Rev. 2024 Nov 16;27(4):1031–53. doi: 10.1007/s10567-024-00507-1 (PMC11609125; doi:10.1007/s10567-024-00507-1)
Supplement: Supplementary file 2 — Supplementary file2 (DOCX 58 KB) [file 10567_2024_507_MOESM2_ESM.docx]

Excluded References

Adamson, L. B., Bakeman, R., Suma, K., & Robins, D. L. (2019). An expanded view of joint attention: Skill, engagement, and language in typical development and autism. *Child Development*, *90*(1), e1–e18. APA PsycInfo®. <https://doi.org/10.1111/cdev.12973>

Allam, H., ElDine, N. G., & Helmy, G. (2008). Scalp acupuncture effect on language development in children with autism: A pilot study. *Journal of Alternative and Complementary Medicine (New York, N.Y.)*, *14*(2), 109–114. <https://doi.org/10.1089/acm.2007.0508>

Alolaby, R. R., Jiraanont, P., Durbin-Johnson, B., Jasoliya, M., Tang, H.-T., Hagerman, R., & Tassone, F. (2020). Molecular Biomarkers Predictive of Sertraline Treatment Response in Young Children With Autism Spectrum Disorder. *Frontiers in Genetics*, *11*, 308. <https://doi.org/10.3389/fgene.2020.00308>

Armstrong, R., Whitehouse, A. J. O., Scott, J. G., Copland, D. A., McMahon, K. L., Fleming, S., & Arnott, W. (2017). A Relationship between Early Language Skills and Adult Autistic-Like Traits: Evidence from a Longitudinal Population-Based Study. *Journal of Autism and Developmental Disorders*, *47*(5), 1478–1489. ERIC. <https://doi.org/10.1007/s10803-016-3014-z>

Arutiunian, V., Lopukhina, A., Minnigulova, A., Shlyakhova, A., Davydova, E., Pereverzeva, D., Sorokin, A., Tyushkevich, S., Mamokhina, U., Danilina, K., & Dragoy, O. (2021). Expressive and Receptive Language in Russian Primary-School-Aged Children with Autism Spectrum Disorder. *Research in Developmental Disabilities*, *117*, 104042. <https://doi.org/10.1016/j.ridd.2021.104042>

Bacon, E. C., Osuna, S., Courchesne, E., & Pierce, K. (2019). Naturalistic language sampling to characterize the language abilities of 3-year-olds with autism spectrum disorder. *Autism : The International Journal of Research and Practice*, *23*(3), 699–712. <https://doi.org/10.1177/1362361318766241>

Bang, J., & Nadig, A. (2015). Learning language in autism: Maternal linguistic input contributes to later vocabulary. *Autism Research*, *8*(2), 214–223. APA PsycInfo®. <https://doi.org/10.1002/aur.1440>

Becerra-Culqui, T. A., Lynch, F. L., Owen-Smith, A. A., Spitzer, J., & Croen, L. A. (2018). Parental First Concerns and Timing of Autism Spectrum Disorder Diagnosis. *Journal of Autism and Developmental Disorders*, *48*(10), 3367–3376. ERIC. <https://doi.org/10.1007/s10803-018-3598-6>

Bedford, R., Gliga, T., Frame, K., Hudry, K., Chandler, S., Johnson, M. H., & Charman, T. (2013). Failure to Learn from Feedback underlies Word Learning Difficulties in Toddlers at Risk for Autism. *Journal of Child Language*, *40*, 29–46. ERIC. <https://doi.org/10.1017/S0305000912000086>

Bedford, R., Pickles, A., & Lord, C. (2016). Early gross motor skills predict the subsequent development of language in children with autism spectrum disorder. *Autism Research : Official Journal of the International Society for Autism Research*, *9*(9), 993–1001. <https://doi.org/10.1002/aur.1587>

Bennett, T. A., Szatmari, P., Bryson, S., Duku, E., Vaccarella, L., & Tuff, L. (2013). Theory of mind, language and adaptive functioning in ASD: A neuroconstructivist perspective. *Journal of the Canadian Academy of Child and Adolescent Psychiatry / Journal de l’Académie Canadienne de Psychiatrie de l’enfant et de l’adolescent*, *22*(1), 13–19. APA PsycInfo®.

Bennett, T. A., Szatmari, P., Georgiades, K., Hanna, S., Janus, M., Georgiades, S., Duku, E., Bryson, S., Fombonne, E., Smith, I. M., Mirenda, P., Volden, J., Waddell, C., Roberts, W., Vaillancourt, T., Zwaigenbaum, L., Elsabbagh, M., & Thompson, A. (2015). Do Reciprocal Associations Exist between Social and Language Pathways in Preschoolers with Autism Spectrum Disorders? *Journal of Child Psychology and Psychiatry*, *56*(8), 874–883. ERIC. <https://doi.org/10.1111/jcpp.12356>

Bennett, T., Szatmari, P., Bryson, S., Volden, J., Zwaigenbaum, L., Vaccarella, L., Duku, E., & Boyle, M. (2008). Differentiating autism and Asperger syndrome on the basis of language delay or impairment. *Journal of Autism and Developmental Disorders*, *38*(4), 616–625. APA PsycInfo®. <https://doi.org/10.1007/s10803-007-0428-7>

Bloch, J., Gersten, E., & Kornblum, S. (1980). Evaluation of a language program for young autistic children. *The Journal of Speech and Hearing Disorders*, *45*(1), 76–89. <https://doi.org/10.1044/jshd.4501.76>

Blume, J., Wittke, K., Naigles, L., & Mastergeorge, A. M. (2021). Language growth in young children with autism: Interactions between language production and social communication. *Journal of Autism and Developmental Disorders*, *51*(2), 644–665. APA PsycInfo®. <https://doi.org/10.1007/s10803-020-04576-3>

Bono, M. A., Daley, T., & Sigman, M. (2004). Relations among Joint Attention, Amount of Intervention and Language Gain in Autism. *Journal of Autism and Developmental Disorders*, *34*(5), 495–505. ERIC. <https://doi.org/10.1007/s10803-004-2545-x>

Bopp, K. D., & Mirenda, P. (2011). Prelinguistic predictors of language development in children with autism spectrum disorders over four-five years. *Journal of Child Language*, *38*(3), 485–503. <https://doi.org/10.1017/S0305000910000140>

Bopp, K. D., Mirenda, P., & Zumbo, B. D. (2009). Behavior predictors of language development over 2 years in children with autism spectrum disorders. *Journal of Speech, Language, and Hearing Research*, *52*(5), 1106–1120. APA PsycInfo®. <https://doi.org/10.1044/1092-4388(2009/07-0262)>

Bornstein, M. H., Hahn, C.-S., Putnick, D. L., & Pearson, R. M. (2018). Stability of core language skill from infancy to adolescence in typical and atypical development. *Science Advances*, *4*(11), eaat7422. <https://doi.org/10.1126/sciadv.aat7422>

Bottema-Beutel, K., Kim, S. Y., Crowley, S., & Yoder, P. J. (2021). Developmental associations between joint engagement and autistic children’s vocabulary: A cross-lagged panel analysis. *Autism*, *25*(2), 566–575. APA PsycInfo®. <https://doi.org/10.1177/1362361320968641>

Bottema-Beutel, K., Woynaroski, T., Louick, R., Stringer Keefe, E., Watson, L. R., & Yoder, P. J. (2019). Longitudinal associations across vocabulary modalities in children with autism and typical development. *Autism : The International Journal of Research and Practice*, *23*(2), 424–435. <https://doi.org/10.1177/1362361317745856>

Bottema-Beutel, K., Yoder, P. J., Hochman, J. M., & Watson, L. R. (2014). The role of supported joint engagement and parent utterances in language and social communication development in children with autism spectrum disorder. *Journal of Autism and Developmental Disorders*, *44*(9), 2162–2174. APA PsycInfo®. <https://doi.org/10.1007/s10803-014-2092-z>

Brignell, A., May, T., Morgan, A. T., & Williams, K. (2019). Predictors and growth in receptive vocabulary from 4 to 8 years in children with and without autism spectrum disorder: A population-based study. *Autism*, *23*(5), 1322–1334. APA PsycInfo®. <https://doi.org/10.1177/1362361318801617>

Brignell, A., Williams, K., Jachno, K., Prior, M., Reilly, S., & Morgan, A. T. (2018). Patterns and predictors of language development from 4 to 7 years in verbal children with and without autism spectrum disorder. *Journal of Autism and Developmental Disorders*, *48*(10), 3282–3295. APA PsycInfo®. <https://doi.org/10.1007/s10803-018-3565-2>

Brignell, A., Williams, K., Prior, M., Donath, S., Reilly, S., Bavin, E. L., Eadie, P., & Morgan, A. T. (2017). Parent-Reported Patterns of Loss and Gain in Communication in 1- to 2-Year-Old Children Are Not Unique to Autism Spectrum Disorder. *Autism: The International Journal of Research and Practice*, *21*(3), 344–356. ERIC. <https://doi.org/10.1177/1362361316644729>

Brito, A. R., Vairo, G. de P. T., Dias, A. P. B. H., Olej, B., Nascimento, O. J. M., & Vasconcelos, M. M. (2021). Effect of prednisolone on language function in children with autistic spectrum disorder: A randomized clinical trial. *Jornal de Pediatria*, *97*(1), 22–29. <https://doi.org/10.1016/j.jped.2019.10.012>

Bruyneel, E., Demurie, E., Warreyn, P., Beyers, W., Boterberg, S., Bontinck, C., Dewaele, N., & Roeyers, H. (2019). Language growth in very young siblings at risk for autism spectrum disorder. *International Journal of Language & Communication Disorders*, *54*(6), 940–953. <https://doi.org/10.1111/1460-6984.12497>

Bruyneel, E., Demurie, E., Warreyn, P., & Roeyers, H. (2019). The mediating role of joint attention in the relationship between motor skills and receptive and expressive language in siblings at risk for autism spectrum  disorder. *Infant Behavior & Development*, *57*, 101377. <https://doi.org/10.1016/j.infbeh.2019.101377>

Buzhardt, J., Wallisch, A., Irvin, D., Boyd, B., Salley, B., & Jia, F. (2022). Exploring Growth in Expressive Communication of Infants and Toddlers With Autism Spectrum Disorder. *Journal of Early Intervention*, *44*(1), 3–22. <https://doi.org/10.1177/1053815121995578>

Cadigan, K., & Missall, K. N. (2007). Measuring Expressive Language Growth in Young Children with Autism Spectrum Disorders. *Topics in Early Childhood Special Education*, *27*(2), 110–118. ERIC.

Cantwell, D. P., Baker, L., Rutter, M., & Mawhood, L. (1989). Infantile autism and developmental receptive dysphasia: A comparative follow-up into middle childhood. *Journal of Autism and Developmental Disorders*, *19*(1), 19–31. APA PsycInfo®. <https://doi.org/10.1007/BF02212715>

Cantwell, D. P., Howlin, P., & Rutter, M. (1977). The analysis of language level and language function: A methodological study. *British Journal of Disorders of Communication*, *12*(2), 119–135. APA PsycInfo®. <https://doi.org/10.3109/13682827709011316>

Casenhiser, D. M., Binns, A., McGill, F., Morderer, O., & Shanker, S. G. (2015). Measuring and Supporting Language Function for Children with Autism: Evidence from a Randomized Control Trial of a Social-Interaction-Based Therapy. *Journal of Autism and Developmental Disorders*, *45*(3), 846–857. ERIC. <https://doi.org/10.1007/s10803-014-2242-3>

Casenhiser, D. M., Shanker, S. G., & Stieben, J. (2013). Learning through interaction in children with autism: Preliminary data from asocial-communication-based intervention. *Autism : The International Journal of Research and Practice*, *17*(2), 220–241. <https://doi.org/10.1177/1362361311422052>

Charman, T., Baron-Cohen, S., Swettenham, J., Baird, G., Drew, A., & Cox, A. (2003). Predicting Language Outcome in Infants with Autism and Pervasive Developmental Disorder. *International Journal of Language & Communication Disorders*, *38*(3), 265–285. ERIC.

Charman, T., Drew, A., Baird, C., & Baird, G. (2003). Measuring early language development in preschool children with autism spectrum disorder using the MacArthur Communicative Development Inventory (Infant Form). *Journal of Child Language*, *30*(1), 213–236. APA PsycInfo®. <https://doi.org/10.1017/S0305000902005482>

Chawarska, K., Klin, A., Paul, R., & Volkmar, F. (2007). Autism Spectrum Disorder in the Second Year: Stability and Change in Syndrome Expression. *Journal of Child Psychology and Psychiatry*, *48*(2), 128–138. ERIC. <https://doi.org/10.1111/j.1469-7610.2006.01685.x>

Chenausky, K., Nelson, C. 3rd, & Tager-Flusberg, H. (2017). Vocalization Rate and Consonant Production in Toddlers at High and Low Risk for Autism. *Journal of Speech, Language, and Hearing Research : JSLHR*, *60*(4), 865–876. <https://doi.org/10.1044/2016_JSLHR-S-15-0400>

Chenausky, K., Norton, A., Tager-Flusberg, H., & Schlaug, G. (2018). Behavioral predictors of improved speech output in minimally verbal children with autism. *Autism Research : Official Journal of the International Society for Autism Research*, *11*(10), 1356–1365. <https://doi.org/10.1002/aur.2006>

Chericoni, N., de Brito Wanderley, D., Costanzo, V., Diniz-Gonçalves, A., Gille, M. L., Parlato, E., Cohen, D., Apicella, F., Calderoni, S., & Muratori, F. (2016). Pre-linguistic vocal trajectories at 6–18 months of age as early markers of autism. *Frontiers in Psychology*, *7*, 11. APA PsycInfo®.

Chiat, S., & Roy, P. (2013). Early predictors of language and social communication impairments at ages 9-11 years: A follow-up study of early-referred children. *Journal of Speech, Language, and Hearing Research : JSLHR*, *56*(6), 1824–1836. <https://doi.org/10.1044/1092-4388(2013/12-0249)>

Chita-Tegmark, M., Arunachalam, S., Nelson, C. A., & Tager-Flusberg, H. (2015). Eye-tracking measurements of language processing: Developmental differences in children at high risk for ASD. *Journal of Autism and Developmental Disorders*, *45*(10), 3327–3338. <https://doi.org/10.1007/s10803-015-2495-5>

Choi, B., Leech, K. A., Tager-Flusberg, H., & Nelson, C. A. (2018). Development of fine motor skills is associated with expressive language outcomes in infants at high and low risk for autism spectrum disorder. *Journal of Neurodevelopmental Disorders*, *10*(1), 14. <https://doi.org/10.1186/s11689-018-9231-3>

Choi, B., Shah, P., Rowe, M. L., Nelson, C. A., & Tager-Flusberg, H. (2021). A longitudinal study of parent gestures, infant responsiveness, and vocabulary development in infants at risk for autism spectrum disorder. *Journal of Autism and Developmental Disorders*, *51*(11), 3946–3958. APA PsycInfo®. <https://doi.org/10.1007/s10803-020-04855-z>

Clark-Whitney, E., Klein, C. B., Hadley, P. A., Lord, C., & Kim, S. H. (2022). Caregiver Language Input Supports Sentence Diversity in Young Children With Autism Spectrum Disorder. *Journal of Speech, Language, and Hearing Research : JSLHR*, *65*(4), 1465–1477. <https://doi.org/10.1044/2021_JSLHR-21-00458>

Crandall, M. C., Bottema-Beutel, K., McDaniel, J., Watson, L. R., & Yoder, P. J. (2019). Children with Autism Spectrum Disorder May Learn from Caregiver Verb Input Better in Certain Engagement States. *Journal of Autism and Developmental Disorders*, *49*(8), 3102–3112. <https://doi.org/10.1007/s10803-019-04041-w>

Crandall, M. C., McDaniel, J., Watson, L. R., & Yoder, P. J. (2019). The Relation Between Early Parent Verb Input and Later Expressive Verb Vocabulary in Children With Autism Spectrum Disorder. *Journal of Speech, Language, and Hearing Research : JSLHR*, *62*(6), 1787–1797. <https://doi.org/10.1044/2019_JSLHR-L-18-0081>

Cupples, L., Ching, T. Y. C., Crowe, K., Seeto, M., Leigh, G., Street, L., Day, J., Marnane, V., & Thomson, J. (2014). Outcomes of 3-year-old children with hearing loss and different types of additional disabilities. *Journal of Deaf Studies and Deaf Education*, *19*(1), 20–39. <https://doi.org/10.1093/deafed/ent039>

D’Elia, L., Valeri, G., Sonnino, F., Fontana, I., Mammone, A., & Vicari, S. (2014). A longitudinal study of the teacch program in different settings: The potential benefits of low intensity intervention in preschool children with autism spectrum  disorder. *Journal of Autism and Developmental Disorders*, *44*(3), 615–626. <https://doi.org/10.1007/s10803-013-1911-y>

DeLong, G. R., Teague, L. A., & McSwain Kamran, M. (1998). Effects of fluoxetine treatment in young children with idiopathic autism. *Developmental Medicine and Child Neurology*, *40*(8), 551–562. <https://doi.org/10.1111/j.1469-8749.1998.tb15414.x>

Dewrang, P., & Sandberg, A. D. (2010). Parental Retrospective Assessment of Development and Behavior in Asperger Syndrome during the First 2 Years of Life. *Research in Autism Spectrum Disorders*, *4*(3), 461–473. ERIC. <https://doi.org/10.1016/j.rasd.2009.11.003>

Dimitrova, N., Özçalışkan, Ş., & Adamson, L. B. (2016). Parents’ Translations of Child Gesture Facilitate Word Learning in Children with Autism, Down Syndrome and Typical Development. *Journal of Autism and Developmental Disorders*, *46*(1), 221–231. <https://doi.org/10.1007/s10803-015-2566-7>

Dimitrova, N., Özçalışkan, Ş., & Adamson, L. B. (2018). "Parents’ translations of child gesture facilitate word learning in children with autism, Down syndrome and typical development": Correction. *Journal of Autism and Developmental Disorders*, *48*(2), 637. APA PsycInfo®. <https://doi.org/10.1007/s10803-017-3346-3>

DiStefano, C., Shih, W., Kaiser, A., Landa, R., & Kasari, C. (2016). Communication growth in minimally verbal children with ASD: The importance of interaction. *Autism Research : Official Journal of the International Society for Autism Research*, *9*(10), 1093–1102. <https://doi.org/10.1002/aur.1594>

Drew, A., Baird, G., Baron-Cohen, S., Cox, A., Slonims, V., Wheelwright, S., Swettenham, J., Berry, B., & Charman, T. (2002). A pilot randomised control trial of a parent training intervention for pre-school children with autism. Preliminary findings and methodological challenges. *European Child & Adolescent Psychiatry*, *11*(6), 266–272. <https://doi.org/10.1007/s00787-002-0299-6>

Droucker, D., Curtin, S., & Vouloumanos, A. (2013). Linking Infant-Directed Speech and Face Preferences to Language Outcomes in Infants at Risk for Autism Spectrum Disorder. *Journal of Speech, Language, and Hearing Research*, *56*(2), 567–576. <https://doi.org/10.1044/1092-4388(2012/11-0266)>

Durrleman, S., Burnel, M., & Reboul, A. (2017). Theory of mind in SLI revisited: Links with syntax, comparisons with ASD. *International Journal of Language & Communication Disorders*, *52*(6), 816–830. <https://doi.org/10.1111/1460-6984.12317>

Dworzynski, K., Ronald, A., Hayiou-Thomas, M. E., McEwan, F., Happe, F., Bolton, P., & Plomin, R. (2008). Developmental Path between Language and Autistic-Like Impairments: A Twin Study. *Infant and Child Development*, *17*(2), 121–136. ERIC. <https://doi.org/10.1002/icd.536>

Dworzynski, K., Ronald, A., Hayiou-Thomas, M., Rijsdijk, F., Happé, F., Bolton, P. F., & Plomin, R. (2007). Aetiological relationship between language performance and autistic-like traits in childhood: A twin study. *International Journal of Language & Communication Disorders*, *42*(3), 273–292. <https://doi.org/10.1080/13682820600939002>

Dykstra, J. R., Sabatos-DeVito, M. G., Irvin, D. W., Boyd, B. A., Hume, K. A., & Odom, S. L. (2013). Using the Language Environment Analysis (LENA) System in Preschool Classrooms with Children with Autism Spectrum Disorders. *Autism: The International Journal of Research and Practice*, *17*(5), 582–594. ERIC. <https://doi.org/10.1177/1362361312446206>

Edmunds, S. R., Ibañez, L. V., Warren, Z., Messinger, D. S., & Stone, W. L. (2017). Longitudinal prediction of language emergence in infants at high and low risk for autism spectrum disorder. *Development and Psychopathology*, *29*(1), 319–329. <https://doi.org/10.1017/S0954579416000146>

Elsabbagh, M., Bedford, R., Senju, A., Charman, T., Pickles, A., & Johnson, M. H. (2014). What you see is what you get: Contextual modulation of face scanning in typical and atypical development. *Social Cognitive and Affective Neuroscience*, *9*(4), 538–543. <https://doi.org/10.1093/scan/nst012>

Fasano, R. M., Perry, L. K., Zhang, Y., Vitale, L., Wang, J., Song, C., & Messinger, D. S. (2021). A granular perspective on inclusion: Objectively measured interactions of preschoolers with and without autism. *Autism Research : Official Journal of the International Society for Autism Research*, *14*(8), 1658–1669. <https://doi.org/10.1002/aur.2526>

Ference, J., & Curtin, S. (2015). The ability to map differentially stressed labels to objects predicts language development at 24 months in 12‐month‐olds at high risk for autism. *Infancy*, *20*(3), 242–262. APA PsycInfo®. <https://doi.org/10.1111/infa.12074>

Flippin, M., & Watson, L. R. (2015). Fathers’ and mothers’ verbal responsiveness and the language skills of young children with autism spectrum disorder. *American Journal of Speech-Language Pathology*, *24*(3), 400–410. APA PsycInfo®. <https://doi.org/10.1044/2015_AJSLP-13-0138>

Franchini, M., Duku, E., Armstrong, V., Brian, J., Bryson, S. E., Garon, N., Roberts, W., Roncadin, C., Zwaigenbaum, L., & Smith, I. M. (2018). Variability in Verbal and Nonverbal Communication in Infants at Risk for Autism Spectrum Disorder: Predictors and Outcomes. *Journal of Autism and Developmental Disorders*, *48*(10), 3417–3431. <https://doi.org/10.1007/s10803-018-3607-9>

Frazier, T. W., Klingemier, E. W., Anderson, C. J., Gengoux, G. W., Youngstrom, E. A., & Hardan, A. Y. (2021). A longitudinal study of language trajectories and treatment outcomes of early intensive behavioral intervention for autism. *Journal of Autism and Developmental Disorders*, *51*(12), 4534–4550. APA PsycInfo®. <https://doi.org/10.1007/s10803-021-04900-5>

Frost, K. M., Pomales-Ramos, A., & Ingersoll, B. (2022). Brief Report: Response to Joint Attention and Object Imitation as Predictors of Expressive and Receptive Language Growth Rate in Young Children on the Autism  Spectrum. *Journal of Autism and Developmental Disorders*. <https://doi.org/10.1007/s10803-022-05567-2>

Fusaroli, R., Weed, E., Fein, D., & Naigles, L. (2019). Hearing me hearing you: Reciprocal effects between child and parent language in autism and typical development. *Cognition*, *183*, 1–18. <https://doi.org/10.1016/j.cognition.2018.10.022>

Gagnon, D., Zeribi, A., Douard, É., Courchesne, V., Rodríguez-Herreros, B., Huguet, G., Jacquemont, S., Loum, M. A., & Mottron, L. (2021). Bayonet-shaped language development in autism with regression: A retrospective study. *Molecular Autism*, *12*(1), 35. <https://doi.org/10.1186/s13229-021-00444-8>

Gamliel, I., Yirmiya, N., & Sigman, M. (2007). The Development of Young Siblings of Children with Autism from 4 to 54 Months. *Journal of Autism and Developmental Disorders*, *37*(1), 171–183. ERIC. <https://doi.org/10.1007/s10803-006-0341-5>

Gengoux, G. W., Schapp, S., Burton, S., Ardel, C. M., Libove, R. A., Baldi, G., Berquist, K. L., Phillips, J. M., & Hardan, A. Y. (2019). Effects of a parent-implemented Developmental Reciprocity Treatment Program for children with autism spectrum disorder. *Autism : The International Journal of Research and Practice*, *23*(3), 713–725. <https://doi.org/10.1177/1362361318775538>

Geoffray, M.-M., Denis, A., Mengarelli, F., Peter, C., Gallifet, N., Beaujeard, V., Grosmaitre, C. J., Malo, V., Grisi, S., Georgieff, N., Magnificat, S., & Touzet, S. (2019). Using ESDM 12 hours per week in children with autism spectrum disorder: Feasibility and results of an observational study. *Psychiatria Danubina*, *31*(3), 333–339. <https://doi.org/10.24869/psyd.2019.333>

Glauser, J., Wilkinson, C. L., Gabard-Durnam, L. J., Choi, B., Tager-Flusberg, H., & Nelson, C. A. (2022). Neural correlates of face processing associated with development of social communication in 12-month infants with familial risk of autism spectrum disorder. *Journal of Neurodevelopmental Disorders*, *14*, 14. APA PsycInfo®. <https://doi.org/10.1186/s11689-021-09413-x>

Goldberg, W. A., Osann, K., Filipek, P. A., Laulhere, T., Jarvis, K., Modahl, C., Flodman, P., & Spence, M. A. (2003). Language and other regression: Assessment and timing. *Journal of Autism and Developmental Disorders*, *33*(6), 607–616. APA PsycInfo®. <https://doi.org/10.1023/B:JADD.0000005998.47370.ef>

Goldberg, W. A., Thorsen, K. L., Osann, K., & Spence, M. A. (2008). Use of home videotapes to confirm parental reports of regression in autism. *Journal of Autism and Developmental Disorders*, *38*(6), 1136–1146. <https://doi.org/10.1007/s10803-007-0498-6>

Goodwin, A., Fein, D., & Naigles, L. (2015). The role of maternal input in the development of wh-question comprehension in autism and typical development. *Journal of Child Language*, *42*(1), 32–63. APA PsycInfo®. <https://doi.org/10.1017/S0305000913000524>

Goodwin, A., Matthews, N. L., & Smith, C. J. (2017). The Effects of Early Language on Age at Diagnosis and Functioning at School Age in Children with Autism Spectrum Disorder. *Journal of Autism and Developmental Disorders*, *47*(7), 2176–2188. <https://doi.org/10.1007/s10803-017-3133-1>

Grandgeorge, M., Hausberger, M., Tordjman, S., Deleau, M., Lazartigues, A., & Lemonnier, E. (2009). Environmental factors influence language development in children with autism spectrum disorders. *PloS One*, *4*(4), e4683. <https://doi.org/10.1371/journal.pone.0004683>

Green, J., Charman, T., McConachie, H., Aldred, C., Slonims, V., Howlin, P., Le Couteur, A., Leadbitter, K., Hudry, K., Byford, S., Barrett, B., Temple, K., Macdonald, W., & Pickles, A. (2010). Parent-mediated communication-focused treatment in children with autism (PACT): A randomised controlled trial. *Lancet (London, England)*, *375*(9732), 2152–2160. <https://doi.org/10.1016/S0140-6736(10)60587-9>

Green, J., Pickles, A., Pasco, G., Bedford, R., Wan, M. W., Elsabbagh, M., Slonims, V., Gliga, T., Jones, E., Cheung, C., Charman, T., & Johnson, M. (2017). Randomised trial of a parent-mediated intervention for infants at high risk for autism: Longitudinal outcomes to age 3 years. *Journal of Child Psychology and Psychiatry, and Allied Disciplines*, *58*(12), 1330–1340. <https://doi.org/10.1111/jcpp.12728>

Gulsrud, A. C., Hellemann, G. S., Freeman, S. F. N., & Kasari, C. (2014). Two to ten years: Developmental trajectories of joint attention in children with ASD who received targeted social communication interventions. *Autism Research : Official Journal of the International Society for Autism Research*, *7*(2), 207–215. <https://doi.org/10.1002/aur.1360>

Haebig, E., McDuffie, A., & Weismer, S. E. (2013). The Contribution of Two Categories of Parent Verbal Responsiveness to Later Language for Toddlers and Preschoolers on the Autism Spectrum. *American Journal of Speech-Language Pathology*, *22*(1), 57–70. ERIC. <https://doi.org/10.1044/1058-0360(2012/11-0004)>

Hagberg, B. S., Miniscalco, C., & Gillberg, C. (2010). Clinic Attenders with Autism or Attention-Deficit/Hyperactivity Disorder: Cognitive Profile at School Age and Its Relationship to Preschool Indicators of Language Delay. *Research in Developmental Disabilities: A Multidisciplinary Journal*, *31*(1), 1–8. ERIC. <https://doi.org/10.1016/j.ridd.2009.07.012>

Hale, C. M., & Tager-Flusberg, H. (2005). Social communication in children with autism: The relationship between theory of mind and discourse development. *Autism : The International Journal of Research and Practice*, *9*(2), 157–178. <https://doi.org/10.1177/1362361305051395>

Hansén-Larson, J., Bejnö, H., Jägerskogh, E., Eikeseth, S., & Klintwall, L. (2021). TRAS: Validity and sensitivity of a language assessment tool for children with ASD. *Scandinavian Journal of Psychology*, *62*(4), 522–528. <https://doi.org/10.1111/sjop.12736>

Hao, Y., Franco, J. H., Sundarrajan, M., & Chen, Y. (2021). A Pilot Study Comparing Tele-Therapy and In-Person Therapy: Perspectives from Parent-Mediated Intervention for Children with Autism Spectrum Disorders. *Journal of Autism and Developmental Disorders*, *51*(1), 129–143. ERIC. <https://doi.org/10.1007/s10803-020-04439-x>

Hardan, A. Y., Gengoux, G. W., Berquist, K. L., Libove, R. A., Ardel, C. M., Phillips, J., Frazier, T. W., & Minjarez, M. B. (2015). A randomized controlled trial of pivotal response treatment group for parents of children with autism. *Journal of Child Psychology and Psychiatry*, *56*(8), 884–892. APA PsycInfo®. <https://doi.org/10.1111/jcpp.12354>

Harper, J. (1975). Age and type of onset as critical variables in early infantile autism. *Journal of Autism and Childhood Schizophrenia*, *5*(1), 25–36. <https://doi.org/10.1007/BF01537970>

Harris, S. L. & And Others. (1981). The Acquisition of Language Skills by Autistic Children: Can Parents Do the Job? *Journal of Autism and Developmental Disorders*, *11*(4), 373–384. ERIC.

Harris, S. L., Handleman, J. S., Kristoff, B., Bass, L., & Gordon, R. (1990). Changes in language development among autistic and peer children in segregated and integrated preschool settings. *Journal of Autism and Developmental Disorders*, *20*(1), 23–31. <https://doi.org/10.1007/BF02206854>

Henry, A. R., & Solari, E. J. (2020). Targeting oral language and listening comprehension development for students with autism spectrum disorder: A school-based pilot study. *Journal of Autism and Developmental Disorders*, *50*(10), 3763–3776. APA PsycInfo®. <https://doi.org/10.1007/s10803-020-04434-2>

Howe, Y. J., O’Rourke, J. A., Yatchmink, Y., Viscidi, E. W., Jones, R. N., & Morrow, E. M. (2015). Female Autism Phenotypes Investigated at Different Levels of Language and Developmental Abilities. *Journal of Autism and Developmental Disorders*, *45*(11), 3537–3549. <https://doi.org/10.1007/s10803-015-2501-y>

Howlin, P. (1981). The results of a home-based language training programme with autistic children. *The British Journal of Disorders of Communication*, *16*(2), 73–88. <https://doi.org/10.3109/13682828109011388>

Howlin, P. (1982). Echolalic and spontaneous phrase speech in autistic children. *Journal of Child Psychology and Psychiatry, and Allied Disciplines*, *23*(3), 281–293. <https://doi.org/10.1111/j.1469-7610.1982.tb00073.x>

Hus, V., Taylor, A., & Lord, C. (2011). Telescoping of caregiver report on the Autism Diagnostic Interview—Revised. *Journal of Child Psychology and Psychiatry, and Allied Disciplines*, *52*(7), 753–760. <https://doi.org/10.1111/j.1469-7610.2011.02398.x>

Ingersoll, B. R., & Wainer, A. L. (2013). Pilot study of a school-based parent training program for preschoolers with ASD. *Autism : The International Journal of Research and Practice*, *17*(4), 434–448. <https://doi.org/10.1177/1362361311427155>

Iverson, J. M. (2018). Early Motor and Communicative Development in Infants With an Older Sibling With Autism Spectrum Disorder. *Journal of Speech, Language, and Hearing Research : JSLHR*, *61*(11), 2673–2684. <https://doi.org/10.1044/2018_JSLHR-L-RSAUT-18-0035>

Iverson, J. M., & Wozniak, R. H. (2007). Variation in Vocal-Motor Development in Infant Siblings of Children with Autism. *Journal of Autism and Developmental Disorders*, *37*(1), 158–170. ERIC. <https://doi.org/10.1007/s10803-006-0339-z>

Kasari, C., Paparella, T., Freeman, S., & Jahromi, L. B. (2008). Language outcome in autism: Randomized comparison of joint attention and play interventions. *Journal of Consulting and Clinical Psychology*, *76*(1), 125–137. <https://doi.org/10.1037/0022-006X.76.1.125>

Kasari, C., Siller, M., Huynh, L. N., Shih, W., Swanson, M., Hellemann, G. S., & Sugar, C. A. (2014). Randomized controlled trial of parental responsiveness intervention for toddlers at high risk for autism. *Infant Behavior & Development*, *37*(4), 711–721. <https://doi.org/10.1016/j.infbeh.2014.08.007>

Kim, H., Ahn, J., Lee, H., Ha, S., & Cheon, K. A. (2020). Differences in Language Ability and Emotional-Behavioral Problems according to Symptom Severity in Children with Autism Spectrum Disorder. *Yonsei Medical Journal*, *61*(10), 880–890. <https://doi.org/10.3349/ymj.2020.61.10.880>

Kim, H. U. (2008). Development of early language and motor skills in preschool children with autism. *Perceptual and Motor Skills*, *107*(2), 403–406. APA PsycInfo®. <https://doi.org/10.2466/PMS.107.6.403-406>

Kjellmer, L., Fernell, E., Gillberg, C., & Norrelgen, F. (2018). Speech and language profiles in 4- to 6-year-old children with early diagnosis of autism spectrum disorder without intellectual disability. *Neuropsychiatric Disease and Treatment*, *14*, 13. APA PsycInfo®. <https://doi.org/10.2147/NDT.S171971>

Klein, S. K., Tuchman, R. F., & Rapin, I. (2000). The influence of premorbid language skills and behavior on language recovery in children with verbal auditory agnosia. *Journal of Child Neurology*, *15*(1), 36–43. APA PsycInfo®. <https://doi.org/10.1177/088307380001500109>

Koegel, R. L., Shirotova, L., & Koegel, L. K. (2009). Antecedent Stimulus Control: Using Orienting Cues to Facilitate First-Word Acquisition for Nonresponders with Autism. *Behavior Analyst*, *32*(2), 281–284. ERIC.

Konstantareas, M. M., & Leibovitz, S. F. (1981). Early Communication Acquisition by Autistic Children: Signing & Mouthing vs. Signing & Speaking. In *Sign Language Studies* (63656390; EJ247264; pp. 135–154). ERIC. <https://www.proquest.com/reports/early-communication-acquisition-autistic-children/docview/63656390/se-2?accountid=14512>

Kover, S. T., Edmunds, S. R., & Ellis Weismer, S. (2016). Brief Report: Ages of Language Milestones as Predictors of Developmental Trajectories in Young Children with Autism Spectrum Disorder. *Journal of Autism and Developmental Disorders*, *46*(7), 2501–2507. <https://doi.org/10.1007/s10803-016-2756-y>

LeBarton, E. S., & Iverson, J. M. (2013). Fine motor skill predicts expressive language in infant siblings of children with autism. *Developmental Science*, *16*(6), 815–827. APA PsycInfo®.

LeBarton, E. S., & Landa, R. J. (2019). Infant motor skill predicts later expressive language and autism spectrum disorder diagnosis. *Infant Behavior & Development*, *54*, 37–47. <https://doi.org/10.1016/j.infbeh.2018.11.003>

Lim, H. A. (2010). Effect of “Developmental speech and language training through music” on speech production in children with autism spectrum disorder. *Journal of Music Therapy*, *47*(1), 2–26. APA PsycInfo®. <https://doi.org/10.1093/jmt/47.1.2>

Lim, H. A., & Draper, E. (2011). The effects of music therapy incorporated with applied behavior analysis verbal behavior approach for children with autism spectrum disorders. *Journal of Music Therapy*, *48*(4), 532–550. <https://doi.org/10.1093/jmt/48.4.532>

Lin, P.-I., Chien, Y.-L., Wu, Y.-Y., Chen, C.-H., Gau, S. S.-F., Huang, Y.-S., Liu, S.-K., Tsai, W.-C., & Chiu, Y.-N. (2012). The WNT2 Gene Polymorphism Associated with Speech Delay Inherent to Autism. *Research in Developmental Disabilities: A Multidisciplinary Journal*, *33*(5), 1533–1540. ERIC. <https://doi.org/10.1016/j.ridd.2012.03.004>

Lombardo, M. V., Busuoli, E. M., Schreibman, L., Stahmer, A. C., Pramparo, T., Landi, I., Mandelli, V., Bertelsen, N., Barnes, C. C., Gazestani, V., Lopez, L., Bacon, E. C., Courchesne, E., & Pierce, K. (2021). Pre-treatment clinical and gene expression patterns predict developmental change in early intervention in autism. *Molecular Psychiatry*, *26*(12), 7641–7651. <https://doi.org/10.1038/s41380-021-01239-2>

Lung, F.-W., & Shu, B.-C. (2021). Two-stage window screening and development trajectories in early identification of autism spectrum disorder among Han Chinese children. *BMC Research Notes*, *14*(1), 130. <https://doi.org/10.1186/s13104-021-05548-1>

Malesa, E., Foss-Feig, J., Yoder, P., Warren, Z., Walden, T., & Stone, W. L. (2013). Predicting language and social outcomes at age 5 for later-born siblings of children with autism spectrum disorders. *Autism : The International Journal of Research and Practice*, *17*(5), 558–570. <https://doi.org/10.1177/1362361312444628>

Manwaring, S. S., Mead, D. L., Swineford, L., & Thurm, A. (2017). Modelling gesture use and early language development in autism spectrum disorder. *International Journal of Language & Communication Disorders*, *52*(5), 637–651. APA PsycInfo®. <https://doi.org/10.1111/1460-6984.12308>

Manwaring, S. S., Swineford, L., Mead, D. L., Yeh, C.-C., & Thurm, A. (2019). The gesture–language association over time in toddlers with and without language delays. *Autism & Developmental Language Impairments*, *4*, 15. APA PsycInfo®. <https://doi.org/10.1177/2396941519845545>

Mason, L. L., & Andrews, A. (2021). Referent-based instruction to strengthen the verbal behavior of early learners with autism and related language disorders. *Behavior Analysis in Practice*, *14*(3), 660–672. APA PsycInfo®. <https://doi.org/10.1007/s40617-020-00491-2>

Mawhood, L., Howlin, P., & Rutter, M. (2000). Autism and developmental receptive language disorder—A comparative follow-up in early adult life. I: Cognitive and language outcomes. *Journal of Child Psychology and Psychiatry, and Allied Disciplines*, *41*(5), 547–559. <https://doi.org/10.1111/1469-7610.00642>

Mayo, J., Chlebowski, C., Fein, D. A., & Eigsti, I.-M. (2013). Age of First Words Predicts Cognitive Ability and Adaptive Skills in Children with ASD. *Journal of Autism and Developmental Disorders*, *43*(2), 253–264. ERIC. <https://doi.org/10.1007/s10803-012-1558-0>

McDaniel, J., Woynaroski, T., Keceli-Kaysili, B., Watson, L. R., & Yoder, P. (2019). Vocal Communication With Canonical Syllables Predicts Later Expressive Language Skills in Preschool-Aged Children With Autism Spectrum Disorder. *Journal of Speech, Language, and Hearing Research : JSLHR*, *62*(10), 3826–3833. <https://doi.org/10.1044/2019_JSLHR-L-19-0162>

McDaniel, J., Yoder, P., Crandall, M., Millan, M. E., Ardel, C. M., Gengoux, G. W., & Hardan, A. Y. (2020). Effects of pivotal response treatment on reciprocal vocal contingency in a randomized controlled trial of children with autism spectrum disorder. *Autism : The International Journal of Research and Practice*, *24*(6), 1566–1571. <https://doi.org/10.1177/1362361320903138>

McDaniel, J., Yoder, P., Estes, A., & Rogers, S. J. (2020a). Predicting expressive language from early vocalizations in young children with autism spectrum disorder: Which vocal measure is best? *Journal of Speech, Language, and Hearing Research*, *63*(5), 1509–1520. APA PsycInfo®. <https://doi.org/10.1044/2020_JSLHR-19-00281>

McDaniel, J., Yoder, P., Estes, A., & Rogers, S. J. (2020b). Validity of Vocal Communication and Vocal Complexity in Young Children with Autism Spectrum Disorder. *Journal of Autism and Developmental Disorders*, *50*(1), 224–237. <https://doi.org/10.1007/s10803-019-04248-x>

McDaniel, J., Yoder, P., & Watson, L. R. (2017). A path model of expressive vocabulary skills in initially preverbal preschool children with autism spectrum disorder. *Journal of Autism and Developmental Disorders*, *47*(4), 947–960. APA PsycInfo®. <https://doi.org/10.1007/s10803-016-3016-x>

McDuffie, A., & Yoder, P. (2010). Types of parent verbal responsiveness that predict language in young children with autism spectrum disorder. *Journal of Speech, Language, and Hearing Research : JSLHR*, *53*(4), 1026–1039. <https://doi.org/10.1044/1092-4388(2009/09-0023)>

McDuffie, A., Yoder, P., & Stone, W. (2005). Prelinguistic predictors of vocabulary in young children with Autism spectrum disorders. *Journal of Speech, Language, and Hearing Research*, *48*(5), 1080–1097. APA PsycInfo®. <https://doi.org/10.1044/1092-4388(2005/075)>

Miilher, L. P., & Fernandes, F. D. M. (2009). Pragmatic, lexical and grammatical abilities of autistic spectrum children. *Pro-Fono : Revista de Atualizacao Cientifica*, *21*(4), 309–314. <https://doi.org/10.1590/s0104-56872009000400008>

Miniscalco, C., Rudling, M., Råstam, M., Gillberg, C., & Johnels, J. Å. (2014). Imitation (rather than core language) predicts pragmatic development in young children with ASD: A preliminary longitudinal study using CDI parental reports. *International Journal of Language & Communication Disorders*, *49*(3), 369–375. APA PsycInfo®. <https://doi.org/10.1111/1460-6984.12085>

Minjarez, M. B., Williams, S. E., Mercier, E. M., & Hardan, A. Y. (2011). Pivotal Response Group Treatment Program for Parents of Children with Autism. *Journal of Autism and Developmental Disorders*, *41*(1), 92–101. ERIC. <https://doi.org/10.1007/s10803-010-1027-6>

Mitchell, S., Brian, J., Zwaigenbaum, L., Roberts, W., Szatmari, P., Smith, I., & Bryson, S. (2006). Early Language and Communication Development of Infants Later Diagnosed with Autism Spectrum Disorder. *Journal of Developmental and Behavioral Pediatrics*, *27*(Suppl2), S69–S78. APA PsycInfo®. <https://doi.org/10.1097/00004703-200604002-00004>

Mukaddes, N. M., Kaynak, F. N., Kinali, G., Beşikci, H., & Issever, H. (2004). Psychoeducational treatment of children with autism and reactive attachment disorder. *Autism : The International Journal of Research and Practice*, *8*(1), 101–109. <https://doi.org/10.1177/1362361304040642>

Mundy, P., Sigman, M., & Kasari, C. (1990). A longitudinal study of joint attention and language development in autistic children. *Journal of Autism and Developmental Disorders*, *20*(1), 115–128. APA PsycInfo®. <https://doi.org/10.1007/BF02206861>

Mundy, P., Sigman, M., Ungerer, J., & Sherman, T. (1987). Nonverbal communication and play correlates of language development in autistic children. *Journal of Autism and Developmental Disorders*, *17*(3), 349–364. APA PsycInfo®. <https://doi.org/10.1007/BF01487065>

Naigles, L. R., Kelty, E., Jaffery, R., & Fein, D. (2011). Abstractness and continuity in the syntactic development of young children with autism. *Autism Research : Official Journal of the International Society for Autism Research*, *4*(6), 422–437. <https://doi.org/10.1002/aur.223>

Norrelgen, F., Fernell, E., Eriksson, M., Hedvall, Å., Persson, C., Sjölin, M., Gillberg, C., & Kjellmer, L. (2015). Children with autism spectrum disorders who do not develop phrase speech in the preschool years. *Autism : The International Journal of Research and Practice*, *19*(8), 934–943. <https://doi.org/10.1177/1362361314556782>

Northrup, J. B., & Iverson, J. M. (2015). Vocal Coordination During Early Parent-Infant Interactions Predicts Language Outcome in Infant Siblings of Children with Autism Spectrum Disorder. *Infancy : The Official Journal of the International Society on Infant Studies*, *20*(5), 523–547. <https://doi.org/10.1111/infa.12090>

Nowell, S. W., Watson, L. R., Crais, E. R., Baranek, G. T., Faldowski, R. A., & Turner-Brown, L. (2020). Joint attention and sensory-regulatory features at 13 and 22 months as predictors of preschool language and social-communication outcomes. *Journal of Speech, Language, and Hearing Research*, *63*(9), 3100–3116. APA PsycInfo®. <https://doi.org/10.1044/2020_JSLHR-20-00036>

Noyan Erbaş, A., Özcebe, E., & Cak Esen, T. (2021). Investigation of the effect of Hanen’s “More Than Words” on parental self-efficacy, emotional states, perceived social support, and on communication  skills of children with ASD. *Logopedics, Phoniatrics, Vocology*, *46*(1), 17–27. <https://doi.org/10.1080/14015439.2020.1717601>

Ohashi, J. K., Mirenda, P., Marinova-Todd, S., Hambly, C., Fombonne, E., Szatmari, P., Bryson, S., Roberts, W., Smith, I., Vaillancourt, T., Volden, J., Waddell, C., Zwaigenbaum, L., Georgiades, S., Duku, E., & Thompson, A. (2012). Comparing early language development in monolingual- and bilingual- exposed young children with autism spectrum disorders. *Research in Autism Spectrum Disorders*, *6*(2), 890–897. APA PsycInfo®. <https://doi.org/10.1016/j.rasd.2011.12.002>

Ökcün-Akçamuş, M. Ç., Acarlar, F., Keçeli Kaysili, B., & Alak, G. (2019). Examination of the relationship between gestures and vocabulary in children with autism spectrum disorder at different language stages. *Early Child Development and Care*, *189*(5), 777–791. APA PsycInfo®. <https://doi.org/10.1080/03004430.2017.1344233>

Oki, J., & Cho, K. (1996). [A longitudinal study of three-year-old children with delayed development of language]. *[Hokkaido igaku zasshi] The Hokkaido journal of medical science*, *71*(5), 637–650.

Ornitz, E. M., Guthrie, D., & Farley, A. H. (1977). The early development of autistic children. *Journal of Autism and Childhood Schizophrenia*, *7*(3), 207–229. <https://doi.org/10.1007/BF01538999>

Özçalışkan, Ş., Adamson, L. B., & Dimitrova, N. (2016). Early deictic but not other gestures predict later vocabulary in both typical development and autism. *Autism*, *20*(6), 754–763. APA PsycInfo®. <https://doi.org/10.1177/1362361315605921>

Paparella, T., Goods, K. S., Freeman, S., & Kasari, C. (2011). The emergence of nonverbal joint attention and requesting skills in young children with autism. *Journal of Communication Disorders*, *44*(6), 569–583. <https://doi.org/10.1016/j.jcomdis.2011.08.002>

Parsons, D., Cordier, R., Lee, H., Falkmer, T., & Vaz, S. (2019). A randomised controlled trial of an information communication technology delivered intervention for children with autism spectrum disorder living in regional Australia. *Journal of Autism and Developmental Disorders*, *49*(2), 569–581. APA PsycInfo®. <https://doi.org/10.1007/s10803-018-3734-3>

Parsons, L., Cordier, R., Munro, N., & Joosten, A. (2019). A randomized controlled trial of a play-based, peer-mediated pragmatic language intervention for children with autism. *Frontiers in Psychology*, *10*, 15. APA PsycInfo®. <https://doi.org/10.3389/fpsyg.2019.01960>

Parsons, L., Cordier, R., Munro, N., & Joosten, A. (2020). Peer’s pragmatic language outcomes following a peer-mediated intervention for children with autism: A randomised controlled trial. *Research in Developmental Disabilities*, *99*, 13. APA PsycInfo®. <https://doi.org/10.1016/j.ridd.2020.103591>

Patten, E., Belardi, K., Baranek, G. T., Watson, L. R., Labban, J. D., & Oller, D. K. (2014). Vocal Patterns in Infants with Autism Spectrum Disorder: Canonical Babbling Status and Vocalization Frequency. *Journal of Autism and Developmental Disorders*, *44*(10), 2413–2428. ERIC. <https://doi.org/10.1007/s10803-014-2047-4>

Pecukonis, M., Plesa Skwerer, D., Eggleston, B., Meyer, S., & Tager-Flusberg, H. (2019). Concurrent Social Communication Predictors of Expressive Language in Minimally Verbal Children and Adolescents with Autism Spectrum Disorder. *Journal of Autism and Developmental Disorders*, *49*(9), 3767–3785. <https://doi.org/10.1007/s10803-019-04089-8>

Pecukonis, M., Young, G. S., Brian, J., Charman, T., Chawarska, K., Elsabbagh, M., Iverson, J. M., Jeste, S., Landa, R., Messinger, D. S., Schwichtenberg, A. J., Webb, S. J., Zwaigenbaum, L., & Tager-Flusberg, H. (2022). Early predictors of language skills at 3 years of age vary based on diagnostic outcome: A baby siblings research consortium study. *Autism Research : Official Journal of the International Society for Autism Research*, *15*(7), 1324–1335. <https://doi.org/10.1002/aur.2760>

Perera, H., Jeewandara, K. C., Seneviratne, S., & Guruge, C. (2016). Outcome of Home-Based Early Intervention for Autism in Sri Lanka: Follow-Up of a Cohort and Comparison with a Nonintervention Group. *BioMed Research International*, *2016*, 3284087. <https://doi.org/10.1155/2016/3284087>

Pickles, A., Anderson, D. K., & Lord, C. (2014). Heterogeneity and plasticity in the development of language: A 17-year follow-up of children referred early for possible autism. *Journal of Child Psychology and Psychiatry, and Allied Disciplines*, *55*(12), 1354–1362. <https://doi.org/10.1111/jcpp.12269>

Pickles, A., Simonoff, E., Conti-Ramsden, G., Falcaro, M., Simkin, Z., Charman, T., Chandler, S., Loucas, T., & Baird, G. (2009). Loss of Language in Early Development of Autism and Specific Language Impairment. *Journal of Child Psychology and Psychiatry*, *50*(7), 843–852. ERIC. <https://doi.org/10.1111/j.1469-7610.2008.02032.x>

Pickles, A., Wright, N., Bedford, R., Steiman, M., Duku, E., Bennett, T., Georgiades, S., Kerns, C. M., Mirenda, P., Smith, I. M., Ungar, W. J., Vaillancourt, T., Waddell, C., Zaidman-Zait, A., Zwaigenbaum, L., Szatmari, P., & Elsabbagh, M. (2022). Predictors of language regression and its association with subsequent communication development in children with autism. *Journal of Child Psychology and Psychiatry, and Allied Disciplines*. <https://doi.org/10.1111/jcpp.13565>

Plate, S., Yankowitz, L., Resorla, L., Swanson, M. R., Meera, S. S., Estes, A., Marrus, N., Cola, M., Petrulla, V., Faggen, A., Pandey, J., Paterson, S., Pruett, J. R., Jr., Hazlett, H., Dager, S., St. John, T., Botteron, K., Zwaigenbaum, L., Piven, J., … Parish-Morris, J. (2022). Infant Vocalizing and Phenotypic Outcomes in Autism: Evidence from the First 2 Years. *Child Development*, *93*(2), 468–483. ERIC. <https://doi.org/10.1111/cdev.13697>

Plumb, A. M., & Wetherby, A. M. (2013). Vocalization Development in Toddlers with Autism Spectrum Disorder. *Journal of Speech, Language, and Hearing Research*, *56*(2), 721–734. ERIC. <https://doi.org/10.1044/1092-4388(2012/11-0104)>

Prathanee, B., Lorwatanapongsa, P., Makarabhirom, K., & Wattanawongsawang, W. (2010). Thai speech and language norms for children 2 1/2 to 4 years of age. *Journal of the Medical Association of Thailand = Chotmaihet Thangphaet*, *93 Suppl 4*, S7-15.

Prescott, K. E., & Ellis Weismer, S. (2022). Children with ASD and Communication Regression: Examining Pre-Loss Skills and Later Language Outcomes Through the Preschool Years. *Journal of Autism and Developmental Disorders*, *52*(5), 1956–1970. <https://doi.org/10.1007/s10803-021-05098-2>

Pry, R., Petersen, A., & Baghdadli, A. (2005). The relationship between expressive language level and psychological development in children with autism 5 years of age. *Autism : The International Journal of Research and Practice*, *9*(2), 179–189. <https://doi.org/10.1177/1362361305047222>

Reynolds, L. C., Inder, T. E., Neil, J. J., Pineda, R. G., & Rogers, C. E. (2014). Maternal obesity and increased risk for autism and developmental delay among very preterm infants. *Journal of Perinatology : Official Journal of the California Perinatal Association*, *34*(9), 688–692. <https://doi.org/10.1038/jp.2014.80>

Ringman, J. M., & Jankovic, J. (2000). Occurrence of tics in Asperger’s syndrome and autistic disorder. *Journal of Child Neurology*, *15*(6), 394–400. <https://doi.org/10.1177/088307380001500608>

Riva, V., Caruso, A., Apicella, F., Valeri, G., Vicari, S., Molteni, M., & Scattoni, M. L. (2021). Early developmental trajectories of expressive vocabulary and gesture production in a longitudinal cohort of Italian infants at high‐risk for autism spectrum disorder. *Autism Research*, *14*(7), 1421–1433. APA PsycInfo®. <https://doi.org/10.1002/aur.2493>

Roemer, E. J., Kushner, E. H., & Iverson, J. M. (2022). Joint Engagement, Parent Labels, and Language Development: Examining Everyday Interactions in Infant Siblings of Children with Autism. *Journal of Autism and Developmental Disorders*, *52*(5), 1984–2003. <https://doi.org/10.1007/s10803-021-05099-1>

Roemer, E. J., West, K. L., Northrup, J. B., & Iverson, J. M. (2019). Word comprehension mediates the link between gesture and word production: Examining language development in infant siblings of children with autism spectrum disorder. *Developmental Science*, *22*(3), 1–10. APA PsycInfo®. <https://doi.org/10.1111/desc.12767>

Romeo, R. R., Choi, B., Gabard-Durnam, L. J., Wilkinson, C. L., Levin, A. R., Rowe, M. L., Tager-Flusberg, H., & Nelson, C. A. 3rd. (2022). Parental Language Input Predicts Neuroscillatory Patterns Associated with Language Development in Toddlers at Risk of Autism. *Journal of Autism and Developmental Disorders*, *52*(6), 2717–2731. <https://doi.org/10.1007/s10803-021-05024-6>

Sansavini, A., Guarini, A., Zuccarini, M., Lee, J. Z., Faldella, G., & Iverson, J. M. (2019). Low rates of pointing in 18-month-olds at risk for autism spectrum disorder and extremely preterm infants: A common index of language delay? *Frontiers in Psychology*, *10*, 12. APA PsycInfo®. <https://doi.org/10.3389/fpsyg.2019.02131>

Saul, J., & Norbury, C. (2020). Does phonetic repertoire in minimally verbal autistic preschoolers predict the severity of later expressive language impairment? *Autism*, *24*(5), 1217–1231. APA PsycInfo®. <https://doi.org/10.1177/1362361319898560>

Schneider, H. D., & Hopp, J. P. (2011). The use of the Bilingual Aphasia Test for assessment and transcranial direct current stimulation to modulate language acquisition in minimally verbal children with autism. *Clinical Linguistics & Phonetics*, *25*(6–7), 640–654. APA PsycInfo®. <https://doi.org/10.3109/02699206.2011.570852>

Sheppard, K. W., Boone, K. M., Gracious, B., Klebanoff, M. A., Rogers, L. K., Rausch, J., Bartlett, C., Coury, D. L., & Keim, S. A. (2017). Effect of Omega-3 and -6 Supplementation on Language in Preterm Toddlers Exhibiting Autism Spectrum Disorder Symptoms. *Journal of Autism and Developmental Disorders*, *47*(11), 3358–3369. ERIC. <https://doi.org/10.1007/s10803-017-3249-3>

Shire, S. Y., Shih, W., & Kasari, C. (2018). Brief Report: Caregiver Strategy Implementation-Advancing Spoken Communication in Children Who are Minimally Verbal. *Journal of Autism and Developmental Disorders*, *48*(4), 1228–1234. <https://doi.org/10.1007/s10803-017-3454-0>

Shumway, S., & Wetherby, A. M. (2009). Communicative acts of children with autism spectrum disorders in the second year of life. *Journal of Speech, Language, and Hearing Research : JSLHR*, *52*(5), 1139–1156. <https://doi.org/10.1044/1092-4388(2009/07-0280)>

Sigman, M., Ruskin, E., Arbeile, S., Corona, R., Dissanayake, C., Espinosa, M., Kim, N., López, A., & Zierhut, C. (1999). Continuity and change in the social competence of children with autism, Down syndrome, and developmental delays. *Monographs of the Society for Research in Child Development*, *64*(1), 1–114. <https://doi.org/10.1111/1540-5834.00002>

Siller, M., Hutman, T., & Sigman, M. (2013). A parent-mediated intervention to increase responsive parental behaviors and child communication in children with ASD: a randomized clinical trial. *Journal of Autism and Developmental Disorders*, *43*(3), 540–555. <https://doi.org/10.1007/s10803-012-1584-y>

Siller, M., & Sigman, M. (2002). The behaviors of parents of children with autism predict the subsequent development of their children’s communication. *Journal of Autism and Developmental Disorders*, *32*(2), 77–89. APA PsycInfo®. <https://doi.org/10.1023/A:1014884404276>

Siller, M., & Sigman, M. (2008). Modeling Longitudinal Change in the Language Abilities of Children with Autism: Parent Behaviors and Child Characteristics as Predictors of Change. *Developmental Psychology*, *44*(6), 1691–1704. ERIC.

Silverman, J. M., Smith, C. J., Schmeidler, J., Hollander, E., Lawlor, B. A., Fitzgerald, M., Buxbaum, J. D., Delaney, K., & Galvin, P. (2002). Symptom domains in autism and related conditions: Evidence for familiality. *American Journal of Medical Genetics*, *114*(1), 64–73. <https://doi.org/10.1002/ajmg.10048>

Simpson, K., Keen, D., & Lamb, J. (2015). Teaching Receptive Labelling to Children with Autism Spectrum Disorder: A Comparative Study Using Infant-Directed Song and Infant-Directed Speech. *Journal of Intellectual & Developmental Disability*, *40*(2), 126–136. ERIC. <https://doi.org/10.3109/13668250.2015.1014026>

Smith, V., Mirenda, P., & Zaidman-Zait, A. (2007). Predictors of expressive vocabulary growth in children with autism. *Journal of Speech, Language, and Hearing Research*, *50*(1), 149–160. APA PsycInfo®. <https://doi.org/10.1044/1092-4388(2007/013)>

Song, X.-K., & So, W.-C. (2022). The influence of child-based factors and parental inputs on expressive language abilities in children with autism spectrum disorder. *Autism : The International Journal of Research and Practice*, *26*(6), 1477–1490. <https://doi.org/10.1177/13623613211054597>

Sparaci, L., Northrup, J. B., Capirci, O., & Iverson, J. M. (2018). From Using Tools to Using Language in Infant Siblings of Children with Autism. *Journal of Autism and Developmental Disorders*, *48*(7), 2319–2334. <https://doi.org/10.1007/s10803-018-3477-1>

Stagnitti, K., O’Connor, C., & Sheppard, L. (2012). Impact of the Learn to Play program on play, social competence and language for children aged 5-8 years who attend a specialist school. *Australian Occupational Therapy Journal*, *59*(4), 302–311. <https://doi.org/10.1111/j.1440-1630.2012.01018.x>

Stone, W. L., McMahon, C. R., Yoder, P. J., & Walden, T. A. (2007). Early social-communicative and cognitive development of younger siblings of children with autism spectrum disorders. *Archives of Pediatrics & Adolescent Medicine*, *161*(4), 384–390. <https://doi.org/10.1001/archpedi.161.4.384>

Stone, W. L., Ousley, O. Y., & Littleford, C. D. (1997). Motor imitation in young children with autism: What’s the object? *Journal of Abnormal Child Psychology*, *25*(6), 475–485. <https://doi.org/10.1023/a:1022685731726>

Stone, W. L., & Yoder, P. J. (2001). Predicting spoken language level in children with autism spectrum disorders. *Autism*, *5*(4), 341–361. APA PsycInfo®. <https://doi.org/10.1177/1362361301005004002>

Sulek, R., Smith, J., Bent, C. A., Hudry, K., Trembath, D., Vivanti, G., & Dissanayake, C. (2022). The utility of LENA as an indicator of developmental outcomes for young children with autism. *International Journal of Language & Communication Disorders*, *57*(1), 103–111. <https://doi.org/10.1111/1460-6984.12678>

Swanson, M. R., Donovan, K., Paterson, S., Wolff, J. J., Parish‐Morris, J., Meera, S. S., Watson, L. R., Estes, A. M., Marrus, N., Elison, J. T., Shen, M. D., McNeilly, H. B., MacIntyre, L., Zwaigenbaum, L., St. John, T., Botteron, K., Dager, S., & Piven, J. (2019). Early language exposure supports later language skills in infants with and without autism. *Autism Research*, *12*(12), 1784–1795. APA PsycInfo®. <https://doi.org/10.1002/aur.2163>

Swanson, M. R., Shen, M. D., Wolff, J. J., Boyd, B., Clements, M., Rehg, J., Elison, J. T., Paterson, S., Parish‐Morris, J., Chappell, J. C., Hazlett, H. C., Emerson, R. W., Botteron, K., Pandey, J., Schultz, R. T., Dager, S. R., Zwaigenbaum, L., Estes, A. M., & Piven, J. (2018). Naturalistic language recordings reveal “hypervocal” infants at high familial risk for autism. *Child Development*, *89*(2), e60–e73. APA PsycInfo®. <https://doi.org/10.1111/cdev.12777>

Swanson, M. R., Shen, M. D., Wolff, J. J., Elison, J. T., Emerson, R. W., Styner, M. A., Hazlett, H. C., Truong, K., Watson, L. R., Paterson, S., Marrus, N., Botteron, K. N., Pandey, J., Schultz, R. T., Dager, S. R., Zwaigenbaum, L., Estes, A. M., & Piven, J. (2017). Subcortical Brain and Behavior Phenotypes Differentiate Infants With Autism Versus Language Delay. *Biological Psychiatry. Cognitive Neuroscience and Neuroimaging*, *2*(8), 664–672. <https://doi.org/10.1016/j.bpsc.2017.07.007>

Szatmari, P., Bryson, S., Duku, E., Vaccarella, L., Zwaigenbaum, L., Bennett, T., & Boyle, M. H. (2009). Similar Developmental Trajectories in Autism and Asperger Syndrome: From Early Childhood to Adolescence. *Journal of Child Psychology and Psychiatry*, *50*(12), 1459–1467. ERIC. <https://doi.org/10.1111/j.1469-7610.2009.02123.x>

Szatmari, P., Bryson, S. E., Streiner, D. L., Wilson, F., Archer, L., & Ryerse, C. (2000). Two-year outcome of preschool children with autism or Asperger’s syndrome. *The American Journal of Psychiatry*, *157*(12), 1980–1987. <https://doi.org/10.1176/appi.ajp.157.12.1980>

Tager-Flusberg, H., Calkins, S., Nolin, T., Baumberger, T., Anderson, M., & Chadwick-Dias, A. (1990). A longitudinal study of language acquisition in autistic and Down syndrome children. *Journal of Autism and Developmental Disorders*, *20*(1), 1–21. <https://doi.org/10.1007/BF02206853>

Talbott, M. R., Nelson, C. A., & Tager-Flusberg, H. (2015). Maternal gesture use and language development in infant siblings of children with autism spectrum disorder. *Journal of Autism and Developmental Disorders*, *45*(1), 4–14. APA PsycInfo®. <https://doi.org/10.1007/s10803-013-1820-0>

Talbott, M. R., Young, G. S., Munson, J., Estes, A., Vismara, L. A., & Rogers, S. J. (2020). The developmental sequence and relations between gesture and spoken language in toddlers with autism spectrum disorder. *Child Development*, *91*(3), 743–753. APA PsycInfo®. <https://doi.org/10.1111/cdev.13203>

Tecoulesco, L., Fein, D., & Naigles, L. R. (2021). What categorical induction variability reveals about typical and atypical development. *Journal of Child Language*, *48*(3), 515–540. APA PsycInfo®. <https://doi.org/10.1017/S0305000920000392>

Tek, S., Mesite, L., Fein, D., & Naigles, L. (2014). Longitudinal analyses of expressive language development reveal two distinct language profiles among young children with autism spectrum disorders. *Journal of Autism and Developmental Disorders*, *44*(1), 75–89. APA PsycInfo®. <https://doi.org/10.1007/s10803-013-1853-4>

Thurm, A., Lord, C., Lee, L.-C., & Newschaffer, C. (2007). Predictors of language acquisition in preschool children with autism spectrum disorders. *Journal of Autism and Developmental Disorders*, *37*(9), 1721–1734. APA PsycInfo®. <https://doi.org/10.1007/s10803-006-0300-1>

Tjus, T., Heimann, M., & Nelson, K. E. (2001). Interaction patterns between children and their teachers when using a specific multimedia and communication strategy: Observations from children with autism and mixed intellectual disabilities. *Autism*, *5*(2), 175–187. APA PsycInfo®. <https://doi.org/10.1177/1362361301005002007>

Toth, K., Munson, J., Meltzoff, A. N., & Dawson, G. (2006). Early Predictors of Communication Development in Young Children with Autism Spectrum Disorder: Joint Attention, Imitation, and Toy Play. *Journal of Autism and Developmental Disorders*, *36*(8), 993–1005. ERIC. <https://doi.org/10.1007/s10803-006-0137-7>

Turner, L. M., Stone, W. L., Pozdol, S. L., & Coonrod, E. E. (2006). Follow-Up of Children with Autism Spectrum Disorders from Age 2 to Age 9. *Autism: The International Journal of Research & Practice*, *10*(3), 243–265. ERIC. <https://doi.org/10.1177/1362361306063296>

Van der Paelt, S., Warreyn, P., & Roeyers, H. (2016). Effect of community interventions on social-communicative abilities of preschoolers with autism spectrum disorder. *Developmental Neurorehabilitation*, *19*(3), 162–174. <https://doi.org/10.3109/17518423.2014.933983>

Van Rooijen, R., Ward, E. K., DE Jonge, M., Kemner, C., & Junge, C. (2022). Two-year-olds at elevated risk for ASD can learn novel words from their parents. *Journal of Child Language*, *49*(5), 1052–1063. <https://doi.org/10.1017/S0305000921000428>

VanMeter, L., Fein, D., Morris, R., Waterhouse, L., & Allen, D. (1997). Delay versus deviance in autistic social behavior. *Journal of Autism and Developmental Disorders*, *27*(5), 557–569. <https://doi.org/10.1023/a:1025830110640>

Veness, C., Prior, M., Bavin, E., Eadie, P., Cini, E., & Reilly, S. (2012). Early Indicators of Autism Spectrum Disorders at 12 and 24 Months of Age: A Prospective, Longitudinal Comparative Study. *Autism: The International Journal of Research and Practice*, *16*(2), 163–177. ERIC. <https://doi.org/10.1177/1362361311399936>

Venker, C. E., Bolt, D. M., Meyer, A., Sindberg, H., Weismer, S. E., & Tager-Flusberg, H. (2015). Parent Telegraphic Speech Use and Spoken Language in Preschoolers with ASD. *Journal of Speech, Language, and Hearing Research*, *58*(6), 1733–1746. ERIC. <https://doi.org/10.1044/2015_JSLHR-L-14-0291>

Venker, C. E., Edwards, J., Saffran, J. R., & Weismer, S. E. (2019). Thinking ahead: Incremental language processing is associated with receptive language abilities in preschoolers with Autism Spectrum Disorder. *Journal of Autism and Developmental Disorders*, *49*(3), 1011–1023. APA PsycInfo®. <https://doi.org/10.1007/s10803-018-3778-4>

Venker, C. E., Kover, S. T., & Weismer, S. E. (2016). Brief Report: Fast Mapping Predicts Differences in Concurrent and Later Language Abilities among Children with ASD. *Journal of Autism and Developmental Disorders*, *46*(3), 1118–1123. ERIC. <https://doi.org/10.1007/s10803-015-2644-x>

Venker, C. E., McDuffie, A., Weismer, S. E., & Abbeduto, L. (2012). Increasing verbal responsiveness in parents of children with autism:A pilot study. *Autism*, *16*(6), 568–585. APA PsycInfo®. <https://doi.org/10.1177/1362361311413396>

Wagner, J. B., Luyster, R. J., Moustapha, H., Tager-Flusberg, H., & Nelson, C. A. (2018). Differential Attention to Faces in Infant Siblings of Children with Autism Spectrum Disorder and Associations with Later Social and Language Ability. *International Journal of Behavioral Development*, *42*(1), 83–92. <https://doi.org/10.1177/0165025416673475>

Walton, K. M., & Ingersoll, B. R. (2016). The utility of Thin Slice ratings for predicting language growth in children with autism spectrum disorder. *Autism*, *20*(3), 374–380. APA PsycInfo®. <https://doi.org/10.1177/1362361315584465>

Weisblatt, E. J., Langensiepen, C. S., Cook, B., Dias, C., Plaisted Grant, K., Dhariwal, M., Fairclough, M. S., Friend, S. E., Malone, A. E., Varga-Elmiyeh, B., Rybicki, A., Karanth, P., & Belmonte, M. K. (2019). A tablet computer-assisted motor and language skills training program to promote communication development in children with autism: Development and pilot study. *International Journal of Human-Computer Interaction*, *35*(8), 643–665. APA PsycInfo®. <https://doi.org/10.1080/10447318.2018.1550176>

West, K. L., Leezenbaum, N. B., Northrup, J. B., & Iverson, J. M. (2019). The Relation Between Walking and Language in Infant Siblings of Children With Autism Spectrum Disorder. *Child Development*, *90*(3), e356–e372. <https://doi.org/10.1111/cdev.12980>

Wetherby, A. M., & Prutting, C. A. (1984). Profiles of Communicative and Cognitive-Social Abilities in Autistic Children. *Journal of Speech, Language, and Hearing Research*, *27*(3), 364–377. <https://doi.org/10.1044/jshr.2703.364>

Whalen, C., Moss, D., Ilan, A. B., Vaupel, M., Fielding, P., Macdonald, K., Cernich, S., & Symon, J. (2010). Efficacy of TeachTown: Basics computer-assisted intervention for the Intensive Comprehensive Autism Program in Los Angeles Unified School District. *Autism : The International Journal of Research and Practice*, *14*(3), 179–197. <https://doi.org/10.1177/1362361310363282>

Whitehouse, A. J. O., Varcin, K. J., Alvares, G. A., Barbaro, J., Bent, C., Boutrus, M., Chetcuti, L., Cooper, M. N., Clark, A., Davidson, E., Dimov, S., Dissanayake, C., Doyle, J., Grant, M., Iacono, T., Maybery, M., Pillar, S., Renton, M., Rowbottam, C., … Hudry, K. (2019). Pre-emptive intervention versus treatment as usual for infants showing early behavioural risk signs of autism spectrum disorder: A single-blind, randomised  controlled trial. *The Lancet. Child & Adolescent Health*, *3*(9), 605–615. <https://doi.org/10.1016/S2352-4642(19)30184-1>

Whiteley, P. (2004). Developmental, behavioural and somatic factors in pervasive developmental disorders: Preliminary analysis. *Child: Care, Health and Development*, *30*(1), 5–11. <https://doi.org/10.1111/j.1365-2214.2004.00380.x>

Wickstrom, J., Farmer, C., Green Snyder, L., Mitz, A. R., Sanders, S. J., Bishop, S., & Thurm, A. (2021). Patterns of delay in early gross motor and expressive language milestone attainment in probands with genetic conditions versus idiopathic ASD from SFARI  registries. *Journal of Child Psychology and Psychiatry, and Allied Disciplines*, *62*(11), 1297–1307. <https://doi.org/10.1111/jcpp.13492>

Wilkinson, C. L., Gabard-Durnam, L. J., Kapur, K., Tager-Flusberg, H., Levin, A. R., & Nelson, C. A. (2020). Use of longitudinal EEG measures in estimating language development in infants with and without familial risk for autism spectrum disorder. *Neurobiology of Language (Cambridge, Mass.)*, *1*(1), 33–53. <https://doi.org/10.1162/nol_a_00002>

Wolf, L., & Goldberg, B. (1986). Autistic children grow up: An eight to twenty-four year follow-up study. *Canadian Journal of Psychiatry. Revue Canadienne de Psychiatrie*, *31*(6), 550–556. <https://doi.org/10.1177/070674378603100613>

Wong, V. C. N., & Kwan, Q. K. (2010). Randomized controlled trial for early intervention for autism: A pilot study of the Autism 1-2-3 Project. *Journal of Autism and Developmental Disorders*, *40*(6), 677–688. <https://doi.org/10.1007/s10803-009-0916-z>

Woynaroski, T., Oller, D. K., Keceli-Kaysili, B., Xu, D., Richards, J. A., Gilkerson, J., Gray, S., & Yoder, P. (2017). The stability and validity of automated vocal analysis in preverbal preschoolers with autism spectrum disorder. *Autism Research : Official Journal of the International Society for Autism Research*, *10*(3), 508–519. <https://doi.org/10.1002/aur.1667>

Woynaroski, T., Watson, L., Gardner, E., Newsom, C. R., Keceli-Kaysili, B., & Yoder, P. J. (2016). Early Predictors of Growth in Diversity of Key Consonants Used in Communication in Initially Preverbal Children with Autism Spectrum Disorder. *Journal of Autism and Developmental Disorders*, *46*(3), 1013–1024. <https://doi.org/10.1007/s10803-015-2647-7>

Woynaroski, T., Yoder, P., & Watson, L. R. (2016). Atypical cross‐modal profiles and longitudinal associations between vocabulary scores in initially minimally verbal children with ASD. *Autism Research*, *9*(2), 301–310. APA PsycInfo®. <https://doi.org/10.1002/aur.1516>

Yoder, P. J. (2006). Predicting lexical density growth rate in young children with autism spectrum disorders. *American Journal of Speech-Language Pathology*, *15*(4), 378–388. APA PsycInfo®. <https://doi.org/10.1044/1058-0360(2006/035)>

Yoder, P. J., & Layton, T. L. (1988). Speech following sign language training in autistic children with minimal verbal language. *Journal of Autism and Developmental Disorders*, *18*(2), 217–229. APA PsycInfo®. <https://doi.org/10.1007/BF02211948>

Yoder, P., & Stone, W. L. (2006). A randomized comparison of the effect of two prelinguistic communication interventions on the acquisition of spoken communication in preschoolers with ASD. *Journal of Speech, Language, and Hearing Research*, *49*(4), 698–711. APA PsycInfo®. <https://doi.org/10.1044/1092-4388(2006/051)>

Yoder, P., Watson, L. R., & Lambert, W. (2015). Value-added predictors of expressive and receptive language growth in initially nonverbal preschoolers with autism spectrum disorders. *Journal of Autism and Developmental Disorders*, *45*(5), 1254–1270. APA PsycInfo®. <https://doi.org/10.1007/s10803-014-2286-4>

Yoshioka, M., & Isaka, M. (2012). [Developmental course of patients with Asperger’s disorder followed objectively from infancy]. *No to hattatsu = Brain and development*, *44*(1), 60–65.

Young, G. S., Merin, N., Rogers, S. J., & Ozonoff, S. (2009). Gaze behavior and affect at 6 months: Predicting clinical outcomes and language development in typically developing infants and infants at risk for autism. *Developmental Science*, *12*(5), 798–814. <https://doi.org/10.1111/j.1467-7687.2009.00833.x>

Zelazo, P. R. (1997). Infant-Toddler Information Processing Treatment of Children with Pervasive Developmental Disorder and Autism: Part II. *Infants and Young Children*, *10*(2), 1–13. ERIC.

Zhang, L., Liu, Y., Zhou, Z., Wei, Y., Wang, J., Yang, J., Wu, Y., & Sun, Y. (2019). A follow-up study on the long-term effects of rehabilitation in children with autism spectrum disorders. *NeuroRehabilitation*, *44*(1), 1–7. <https://doi.org/10.3233/NRE-182502>

Zwaigenbaum, L., Bryson, S. E., Brian, J., Smith, I. M., Roberts, W., Szatmari, P., Roncadin, C., Garon, N., & Vaillancourt, T. (2016). Stability of diagnostic assessment for autism spectrum disorder between 18 and 36 months in a high-risk cohort. *Autism Research : Official Journal of the International Society for Autism Research*, *9*(7), 790–800. <https://doi.org/10.1002/aur.1585>

Zwaigenbaum, L., Bryson, S., Rogers, T., Roberts, W., Brian, J., & Szatmari, P. (2005). Behavioral manifestations of autism in the first year of life. *International Journal of Developmental Neuroscience : The Official Journal of the International Society for Developmental Neuroscience*, *23*(2–3), 143–152. <https://doi.org/10.1016/j.ijdevneu.2004.05.001>

Zwaigenbaum, L., Thurm, A., Stone, W., Baranek, G., Bryson, S., Iverson, J., Kau, A., Klin, A., Lord, C., Landa, R., Rogers, S., & Sigman, M. (2007). Studying the Emergence of Autism Spectrum Disorders in High-risk Infants: Methodological and Practical Issues. *Journal of Autism and Developmental Disorders*, *37*(3), 466–480. <https://doi.org/10.1007/s10803-006-0179-x>
